# Functional interaction between endothelin-1 and ZEB1/YAP signaling regulates cellular plasticity and metastasis in high-grade serous ovarian cancer

**DOI:** 10.1186/s13046-022-02317-1

**Published:** 2022-04-28

**Authors:** Rosanna Sestito, Piera Tocci, Celia Roman, Valeriana Di Castro, Anna Bagnato

**Affiliations:** Preclinical Models and New Therapeutic Agents Unit, IRCCS-Regina Elena National Cancer Institute, Rome, Italy

**Keywords:** Ovarian carcinoma, Endothelin A receptor, ZEB1, YAP, AP-1, ILK, Epithelial-to-mesenchymal transition, Metastasis

## Abstract

**Background:**

Epithelial-to-mesenchymal transition (EMT) encompasses a highly dynamic and complex key process which leads to metastatic progression. In high-grade serous ovarian carcinoma (HG-SOC), endothelin-1 (ET-1)/endothelin A receptor (ET_A_R) signaling promotes EMT driving tumor progression. However, the complex nature of intertwined regulatory circuits activated by ET-1 to trigger the metastatic process is not fully elucidated.

**Methods:**

The capacity of ET-1 pathway to guide a critical transcriptional network that is instrumental for metastatic growth was identified in patient-derived HG-SOC cells and cell lines through immunoblotting, q-RT-PCR, co-immunoprecipitation, in situ proximity ligation, luciferase reporter, chromatin immunoprecipitation assays and publicly available databases. Functional assays in HG-SOC cells and HG-SOC xenografts served to test the inhibitory effects of ET-1 receptors (ET-1R) antagonist in vitro and in vivo.

**Results:**

We demonstrated that ET-1/ET_A_R axis promoted the direct physical ZEB1/YAP interaction by inducing their nuclear accumulation in HG-SOC cells. Moreover, ET-1 directed their engagement in a functional transcriptional complex with the potent oncogenic AP-1 factor JUN. This led to the aberrant activation of common target genes, including *EDN1* (ET-1) gene, thereby creating a feed-forward loop that sustained a persistent ET-1/ZEB1 signaling activity. Notably, ET-1-induced Integrin-linked kinase (ILK) signaling mediated the activation of YAP/ZEB1 circuit driving cellular plasticity, invasion and EMT. Of therapeutic interest, treatment of HG-SOC cells with the FDA approved ET-1R antagonist macitentan, targeting YAP and ZEB1-driven signaling, suppressed metastasis in vivo in mice. High gene expression of ET_A_R/ILK/YAP/AP-1/ZEB1 was a strong predictor of poor clinical outcome in serous ovarian cancer patients, indicating the translational relevance of this signature expression.

**Conclusions:**

This study provides novel mechanistic insights of the ET-1R-driven mediators that support the ability of HG-SOC to acquire metastatic traits which include the cooperation of YAP and ZEB1 regulatory circuit paving the way for innovative treatment of metastatic ovarian cancer.

**Supplementary Information:**

The online version contains supplementary material available at 10.1186/s13046-022-02317-1.

## Background

Metastasis represents one of the challenging issues in the management of high-grade serous ovarian carcinoma (HG-SOC), the most common and aggressive ovarian cancer (OC) [1, 2], and effective therapies to specifically target cancer progression are missing, emphasizing the urgent need for developing new strategies for the treatment of this lethal disease.

The program that endows aggressive traits is a highly coordinated plastic process termed epithelial-to-mesenchymal transition (EMT) by which cancer cells acquire invasive abilities necessary to complete all the steps of the metastatic cascade [3–5]. However, detailed mechanistic insights are still lacking to understand the complex transcriptional states during the metastatic growth. Multiple extra-cellular signals can initiate an EMT-related gene expression program through significant cross-talk between co-factors and transcription factors (TF) forming regulatory networks controlling the metastatic cascade [5, 6]. In this regard, it has been widely demonstrated that endothelin-1 (ET-1) axis, including the peptide ligand ET-1 and the two G-protein coupled receptors (GPGR; ET_A_R and ET_B_R), is a potent inducer of EMT by regulating the EMT-TF, such as Snail and zinc finger E-box binding homeobox 1 (ZEB1), that repress epithelial genes and stimulate the expression of mesenchymal components [7–10]. ZEB1 is a prime element of a network of TF controlling EMT by directly repressing E-cadherin [11, 12]. It has been widely demonstrated that ZEB1 can participate in double-negative feedback loops with microRNA (miR)-200 family members, strong inducers of epithelial differentiation [11–13]. Recent evidence in OC has pointed out the role of ET-1/ET_A_R axis in regulating the ZEB1/miR-200 circuitry [10]. In particular, our results indicate that ET_A_R activation by ET-1 promotes OC progression by inducing ZEB1 expression and suppressing miR-200. Despite this evidence, the complex regulatory networks co-opted by ET-1 fostering HG-SOC cells to undergo EMT and metastasis formation remain to be fully elucidated.

We had previously demonstrated that HG-SOC progression also requires the integration of ET-1 signaling with the transcriptional co-activators of the Hippo pathway, Yes-associated protein (YAP) and PDZ-binding domain (TAZ) [14, 15], those were instrumental for tumour initiation and progression in multiple tissue types [16, 17]. Since YAP/TAZ cannot directly bind DNA, TEA domain (TEAD1-4) DNA-binding family are their main intracellular mediators co-occupying chromatin at composite cis-regulatory elements having TEAD motifs [18]. Besides TEAD, YAP cooperates with other oncogenic TF, including activator protein-1 (AP-1), dimer of JUN and FOS proteins, allowing the formation of nuclear complexes promoting tumor growth and metastases [19–22]. Interestingly, recent discoveries demonstrate that YAP can interact with ZEB1, shifting ZEB1 from a repressor to an activator of gene transcription [21, 23–25]. The functional alliance between ZEB1 and YAP promotes the transcription of a common ZEB1/YAP target gene set, which represents a predictor of poor survival, therapy resistance, and increased metastatic risk in breast and pancreatic cancers [21, 23, 25]. However, the possible ZEB1/YAP signaling cross-talk in mediating ET-1-driven metastatic traits has been never investigated. Understanding the mechanism and functional consequences of ZEB1/YAP-triggered phenotypic plasticity is therefore critical to improve cancer therapeutics. In this regard, this study identifies an integrated YAP/AP-1/ZEB1 circuit that is regulated by ET-1 at multiple levels to induce cellular plasticity fostering metastatic progression. Blockade of ET-1R interrupted the protein–protein interaction between ZEB1 and YAP, and suppressed metastasis of HG-SOC in vivo. Our findings provide new insights into the transcriptional machinery activated by ET-1 for HG-SOC metastatic progression and unveil therapeutic strategy for the metastatic ovarian cancer.

## Methods

### Cell cultures and reagents

HG-SOC primary cells were obtained from freshly-isolated ascitic fluid from HG-SOC patients undergoing surgery for ovarian tumor by laparotomy or paracentesis at the Gynecological Oncology of our Institute. The study protocol for tissue collection and clinical information was approved by the institutional review board (IRB) and patients provided written informed consent authorizing the collection and use of the tissue for study purposes. Patient-derived (PD)-HG-SOC cells were isolated and characterized as previously reported [14]. In this study, we employed the early passage PD-HG-SOC PMOV10 cell line, where PM stands for Preclinical Models, OV stands for ovarian serous cancer, and # is the order in which the cell line was established. In addition, we used HG-SOC cell lines, OVCAR-3 (HTB-161) and Kuramochi, which were obtained from American Type Culture Collection (ATCC) and Japanese Collection of Research Bioresources (JCRB) Cell Bank, respectively. Cells were validated by short tandem repeat (STR) profiling. PMOV10 and Kuramochi cells were cultured in RPMI 1640 (Gibco, ThermoFisher Scientific, USA) containing 1% penicillin–streptomycin and 10% fetal bovine serum, whereas OVCAR-3 cells were cultured in RPMI-1640 containing 1% penicillin–streptomycin, 20% fetal bovine serum and 0.01 mg/ml bovine insulin, under a humidified atmosphere of 5% CO2 at 37 °C. Cells were tested routinely for cell proliferation, as well as mycoplasma contamination. Before each experiment, cells were serum starved by incubation in serum-free medium for 24 h. ET-1 was used at 100 nM and was purchased from Sigma-Aldrich (Germany). Macitentan, also known as ACT-064992 or N-(5-[4-bromophenyl]-6-{2-[5-bromopyrimidin-2-yloxy]-ethoxy}-pyrimidin-4-yl)-N′-propylsulfamide, was added 30 min before ET-1 at a dose of 1 μM and was kindly provided by Actelion Pharmaceuticals, Ltd. (Switzerland). BQ123 (Bachem, Switzerland) and BQ788 (Peninsula Laboratories, USA) were added 30 min before ET-1 at a dose of 1 µM.

### Small interfering RNA (siRNA) transfection

For transient knockdown, PMOV10, OVCAR-3, and Kuramochi cells were transfected for 48–72 h with Dharmacon SMARTPool ON-TARGETplus siRNA oligonucleotides specific for ZEB1 (si-ZEB1, L-006564–01-0050), YAP1 (si-YAP, L-012200–00-0050), JUN (si–c-JUN, L-003268–00-0020), or with ON-TARGETplus Non-targeting Control Pool (SCR, D-001810–10) (GE Healthcare Life Sciences, USA). In addition, PMOV10 and Kuramochi cells were transfected with a Negative Control DsiRNA (#51–01-14–04), or with a siRNA pre-designed and validated for ILK (si-ILK, hs.Ri.ILK.13.2), purchased from IDT (USA). siRNAs were used at a final concentration of 50–100 nM and Lipofectamine RNAiMAX transfection reagent (ThermoFisher Scientific) was employed according to the manufacturer’s protocol.

### Immunoblotting (IB) and immunoprecipitation (IP)

NE-PER nuclear and cytoplasmic extraction reagents kit (Thermo Scientific) was used to separate cytoplasmic and nuclear fractions. Whole cell lysates were prepared using a modified RIPA buffer (50 mM Tris–HCl pH 7.4, 250 mM NaCl, 1% Triton X-100, 1% sodium deoxycholate, 0.1% SDS) containing a mixture of protease and phosphatase inhibitors. Protein content of the extracts was determined using Bio-Rad Protein Assay Kit (Bio-Rad, USA). IB for anti-PCNA and anti-tubulin antibodies (Abs) were used as loading control and to assess the purity of the nuclear and cytoplasmic fractions, respectively. IB for β-actin was used as loading control for whole cell lysates. Cell lysates were resolved by SDS/PAGE. Membranes were blocked in TTBS (TBS with 0.1% Tween 20) containing either 5% dry milk or BSA and incubated with primary antibodies (Abs) overnight at 4 °C. All Abs used in IB assays are listed in Additional file 1: Table S1. After washing, the appropriate secondary peroxidase conjugated Abs were added to membranes and incubated for 1 h. For IP, 200 μg of pre-cleared nuclear cell fractions were incubated with anti-YAP (1A12, Cell Signaling Technology), anti-ZEB1 (H3, Santa Cruz Biotechnology, USA), or anti-mouse IgG Isotype Control (ThermoFisher Scientific) Abs and protein G Sepharose 4 Fast Flow beads (Cytiva, Sweden) at 4 °C overnight. The IP and input (3% of the total nuclear extracts) samples were boiled for 5 min in SDS loading buffer, loaded onto pre-casted 10% or 4–20% SDS/PAGE (Bio-Rad), transferred by using Trans-Blot transfer pack (Bio-Rad), and IB with different Abs as before. To obtain clean and specific IB signals of TEAD4 and JUN, which run very close to heavy chain of IgG, we used HRP-conjugated protein A peroxidase (Pierce, ThermoFisher Scientific) instead of HRP-conjugated secondary Abs. Blots were developed with the enhanced chemiluminescence detection system (Clarity Western ECL Substrate Bio-Rad) or LiteAblot turbo extrasensitive chemiluminescent substrate (Euroclone, Italy). IB signals were quantified using ImageJ software (https://imagej.nih.gov/ij/).

### Proximity ligation assay (PLA)

PMOV10 or Kuramochi cells (4 × 10^4^) were seeded in 24-well plate, and after 24 h of starvation, were stimulated with ET-1 for 6 h. Cells were then washed in PBS, fixed with formaldehyde 4% in PBS for 10 min, permeabilized with Triton X-100 0.4% in PBS for 20 min, blocked with BSA 0.5% in PBS for 30 min and stained with anti-ZEB1 (D80D3, 1:20, cat. #3396, Cell Signaling Technology) together with anti-YAP (G-6, 1:20, SC-376830, Santa Cruz Biotechnology) or anti-ZEB1 (H-3, 1:20, sc-515797, Santa Cruz Biotechnology) together with anti-c-JUN (60A8, 1:20, cat. #9165S, Cell Signaling Technology) primary Abs at 4 °C overnight. PLA was performed with the Duolink in situ Detection Reagents Orange (Sigma-Aldrich, USA), according to the manufacturer’s protocol. Anti-mouse PLUS (Sigma-Aldrich) and anti-rabbit MINUS (Sigma-Aldrich) PLA probes were used for 1 h at 37 °C. Then, coverslips were washed in PBS and then incubated with a DNA ligase diluted in Ligation buffer for 30 min at 37 °C. After washing, coverslips were incubated with a DNA polymerase diluted in the amplification buffer for 100 min at 37 °C. Nuclei were stained using 4′,6′-diamidino-2-phenykindole (DAPI). Coverslips were mounted with Vectashield mounting medium for fluorescence (Vector Laboratories Ltd., UK). Fluorescence signals were captured by using a Leica DMIRE2 microscope equipped with a Leica DFC 350FX camera and elaborated by Leica FW4000 deconvolution software (Leica) using an oil 63 × objective. The number of dots per nuclei was quantified using ImageJ software.

### Chromatin immunoprecipitation (ChIP)

Chromatin was extracted from 5 × 10^6^ cells of PMOV10 cells. Briefly, cells were crosslinked with formaldehyde 1% in PBS for 8 min at room temperature. After washing in PBS, chromatin was sheared by sonication, centrifuged and diluted in 50 mM Tris pH 8.0, 0.5% NP-40, 0.2 M NaCl, 0.5 mM EDTA. One-twentieth of the precleared chromatin was used as the input for the ChIP assay. The precleared chromatin was rotated overnight with primary Ab or IgG. The primary Abs used were as follows: anti-ZEB1 (2 µg/µl, clone H3, sc-515797, Santa Cruz Biotechnology), anti-YAP (2 µg/µl, H-125, cat. no. sc-15407, Santa Cruz Biotechnology), anti-c-JUN (2 µg/µl, 60A8, cat. #9165S, Cell Signaling Technology), anti-rabbit IgG Isotype Control (ThermoFisher Scientific), and anti-mouse IgG Isotype Control (ThermoFisher Scientific). The next day, 40 μl of 50% salmon sperm-saturated protein A Sepharose (Cytiva) was added to immune complexes, and the mixtures were rotated at 4 °C for 30 min. The beads were washed with 20 mM Tris pH 8.0, 0.1% SDS, 1% NP-40, 2 mM EDTA, 500 mM NaCl and with 1 × Tris/EDTA. Immune complexes in 1 × Tris/EDTA containing 1% SDS and protein-DNA cross-links were reverted by incubation at 65 °C for 4 h. DNA–protein complexes were digested with Proteinase K at 37 °C for 1 h. DNA was purified through phenol/chloroform extraction, precipitated in ethanol, and resuspended in water. The binding between ZEB1, YAP, and JUN with AP-1 motif or a negative region in the *EDN1* promoter was examined through qPCR by using AmpliTaq polimerase (Applied Biosystems, USA). The primers used are listed in Additional file 2: Table S2.

### Luciferase reporter gene assay

Luciferase assays were carried out in PMOV10 and OVCAR-3 cells (6 × 10^4^) seeded in 12-well plates and transfected with 500 ng of reporter plasmid by using Lipofectamine 2000 (ThermoFisher Scientific), according to manufacturer’s instructions. Transcriptional activity of AP-1 was studied by using a pAP1-Luc Cis-Reporter plasmid (cat. 219,074, Agilent Technologies, USA). To analyze the ZEB1 and ET-1 promoter activities we used a reporter construct containing a 900 bp sequence from ZEB1 promoter, synthesized by TEMA Ricerca (Italy), and a reporter construct containing a 1500 bp sequence from ET-1 promoter, kindly provided by Dr. Z. Zhang (University of California San Diego School of Medicine, La Jolla, Ca), respectively. All plasmids were co-transfected with 250 ng of pCMV-β-galactosidase vector (Promega) and 100 nM of siRNAs as indicated. After 24 h of transfection, cells were stimulated with ET-1 and/or macitentan for additional 24 h. Reporter activity was measured using the Luciferase assay system (Promega) and normalized to β-galactosidase activity.

### RNA extraction and quantitative real-time PCR (qRT-PCR)

Total RNA was isolated using the Trizol (ThermoFisher Scientific), according to the manufacturer’s protocol. RNA integrity was confirmed through agarose gel electrophoresis, and RNA concentration and purity were determined with a Nanodrop 1000 spectrophotometer (Thermo Fisher Scientific). RNA was reversed transcribed using the Wonder RT cDNA Synthesis kit (Euroclone, Italy). The expression of ET-1, CTGF, CYR61, ANKRD1, E-cadherin, N-cadherin, vimentin, ZEB1, and cyclophilin-A mRNA was evaluated by using Luna Universal qPCR Master Mix (New England Biolabs, USA) on QuantStudio 6-Flex (Thermo Fisher Scientific), according to the manufacturer's instructions. The mRNA expression levels were determined by normalizing to cyclophilin-A mRNA expression and expressed as relative mRNA level (2^ΔΔct). Data are presented as means ± SD. Primer sequences are provided in Additional file 2: Table S2.

### ELISA assay

PMOV10 (1 × 10^6^) cells were seeded in 100 mm dish. After 24 h of siRNA transfection cells medium was replaced with serum-free medium containing or not macitentan. After 48 h of incubation, the conditioned media were collected, centrifuged and stored in aliquots at -80 °C. The release of ET-1 was measured with Quantikine ELISA kit (R&D Systems, USA), according to the manufacturer’s instructions. ET-1 was measured in the range of 0–25 pg/ml.

### Chemoinvasion assay

Invasion assays were carried out using Boyden Chambers consisting of transwell filter inserts with 8 μm size polycarbonate membrane (Corning, USA) placed in a 24-well plate and precoated with 50 μl of cultrex (R&D Systems). After 48 h of siRNA transfection, OVCAR-3, PMOV10, or Kuramochi cells (3 × 10^4^) were seeded with serum-free medium in the upper chamber and serum-free medium containing or not ET-1 in combination or not with macitentan was added to the lower chamber. Cells were left to invade overnight at 37 °C. Cells on the upper part of the membrane were scraped using a cotton swab and invaded cells were stained using Diff-Quick kit (Merz-Dade, Switzerland). From every transwell, several images were taken under a phase-contrast with Olympus I × 70 microscope (Olympus Corporation, Japan) at 10 × magnification for Kuramochi cells or at 20 × magnification for PMOV10 and OVCAR-3 cells.

### Vasculogenic mimicry assay

After 48 h of siRNA transfection, OVCAR-3 (3.5 × 10^4^) and Kuramochi (3 × 10^4^) cells were seeded in a 96-well culture plate precoated with 50 μl/well of Cultrex (R&D Systems) and stimulated with serum-free medium or ET-1 in combination or not with macitentan. Cells were left overnight at 37 °C. The day after, tubule-like structure formations were visualized with an inverted microscope with a 20 × magnification. Representative images were captured with a ZOE Fluorescent Cell Imager (BioRad Laboratories). Tube formation was analyzed by using Angiogenesis Analyzer for ImageJ (NIH) measuring the number of nodes and the tube length.

### Animal study

Female athymic (nu + /nu +) mice, 4–6 weeks of age (Charles River Laboratories, Italy) were injected intraperitoneally with 2.5 × 10^6^ viable OVCAR-3 cells following the guidelines for animal experimentation of the Italian Ministry of Health. One weeks after cells injection, OVCAR-3 xenografts were randomized into two different groups of ten mice undergoing the following treatments: control (Ctr; vehicle) vs. macitentan (MAC; 30 mg/kg/oral daily) for 5 weeks. At the end of treatment, all mice were euthanized and intraperitoneal (i.p.) organs throughout the peritoneal cavity (including intestine, mesentery, liver and spleen) were analyzed. The number of visible metastases was counted and the removed i.p. nodules were carefully dissected, frozen and processed for IB analyses. Values represent the mean ± SD of ten mice in each group for OVCAR-3 xenografts from two independent experiments.

### Bioinformatic analyses

The Kaplan–Meier plotter [26] was used to investigate the correlation between the expression of ET_A_R, ILK, ZEB1, YAP, and AP-1 mRNA levels with the prognosis of serous ovarian cancer patients. Overall survival (OS) analysis was performed in the following cohorts of patients: GSE9891, GSE18520, GSE26193, GSE30161, and GSE63885 (523 patients). In parallel, progression-free survival (PFS) analysis was performed in the following cohorts of patients: GSE9891, GSE26193, GSE30161, and GSE63885 (483 patients). The employed gene probes are as follow: 216235_s_at and 204464_s_at for ET_A_R; 201234_at for ILK; 217836_s_at for YAP; 201464_x_at, 201465_s_at, 201466_s_at, 203751_x_at, 214326_x_at, 203752_s_at, 202768_at and 209189_at for AP1; 210875_s_at, 212764_at, 239952_at and 212758_s_at for ZEB1. OC samples were divided into ‘low’ and ‘high’ according to gene mRNA expression using the auto select best cutoff value. Subsequently, OS and PFS for the two groups were compared with a Kaplan–Meier survival plot on the webpage (http://kmplot.com/analysis/index.php?p=service&cancer=ovar).

### Statistical analysis

The significance of KM curves was evaluated by log-rank test, hazard ratio (HR) with 95% confidence intervals. Except for the animal study, each experiment was repeated at least three times. Data are presented as mean ± SD. Statistical analysis was performed using Student’s two tailed t-test to compare two groups of independent samples. Statistical tests were carried out using GraphPad Prism 8 software (San Diego). *P* < 0.05 was considered statistically significant.

## Results

### ET-1/ETAR axis induces the ZEB1/YAP interaction in HG-SOC cells

**Fig. 1:**
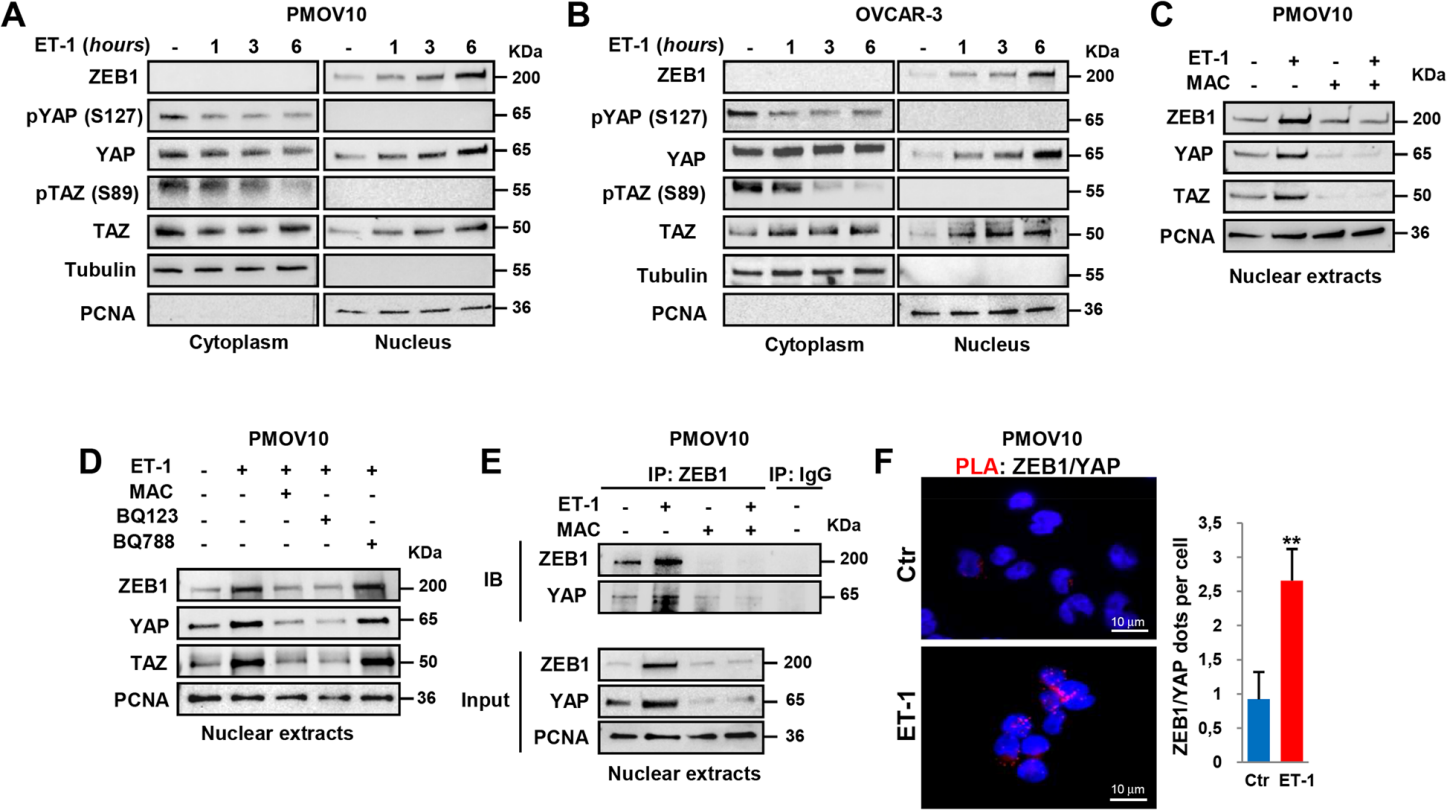
ET-1/ET_A_R axis induces the ZEB1/YAP interaction in HG-SOC cells. **A**, **B** Immunoblotting (IB) analysis of ZEB1, pYAP (S127), YAP, pTAZ (S89) and TAZ protein expression in the cytoplasmic and nuclear extracts of patient-derived HG-SOC PMOV10 (**A**) and OVCAR-3 (**B**) cells stimulated with ET-1 (100 nM) for the indicated times. Tubulin and PCNA were used as cytoplasmic and nuclear loading control, respectively. **C** Nuclear extracts of PMOV10 cells stimulated for 6 h with ET-1 and/or with macitentan (MAC, 1µM), a dual ET-1 receptor antagonist, were IB for ZEB1, YAP, and TAZ. PCNA was used loading control. **D** Nuclear extracts of PMOV10 cells stimulated for 6 h with ET-1 and/or with the selective ET_A_R antagonist BQ123 (1µM), the selective ET_B_R antagonist BQ788 (1µM), or macitentan (1µM), were IB for ZEB1, YAP and TAZ. PCNA was used loading control. **E** Nuclear extracts of PMOV10 cells stimulated as in *C* were immunoprecipitated (IP) for endogenous ZEB1 using anti-ZEB1 antibody (Ab) or anti-immunoglobulin G (IgG) Ab as control and IB using anti-ZEB1 and anti-YAP Abs. PCNA was used as loading control. **F** Representative images of proximity ligation assay (PLA) detection of direct protein–protein interaction between ZEB1 and YAP (red signals) in PMOV10 cells stimulated or not with ET-1 for 6 h. DAPI staining (blue) highlights the nucleus (Magnification: 63x; scale bar: 10 μm). Right graph represents the quantification of the ZEB1/YAP protein interaction. Bars are means ± SD (*n* = 3; **p < 0.01)

Given that ET-1/ET-1R axis is able to induce ZEB1 expression in HG-SOC cells [10] and, in parallel, to promote YAP/TAZ nuclear accumulation [14], we evaluated the potential role of ET-1 signaling to drive the concomitant ZEB1 and YAP/TAZ activation. To this end, we used patient-derived (PD)-HG-SOC PMOV10 cells which, recapitulating the molecular and histological HG-SOC patient features [14], represent a more reliable model for design of targeted therapy for women affected by HG-SOC. In parallel, OVCAR-3 and Kuramochi HG-SOC cell lines were used. Upon ET-1 stimulation, at different time-points, a decrease of YAP/TAZ phosphorylation in the cytoplasm compartment was observed along with a shutting of YAP/TAZ to the nuclear compartment was observed (Fig. 1A, B). Of note, ET-1 stimulation up-regulated ZEB1 nuclear expression with a similar kinetic of YAP/TAZ accumulation (Fig. 1A, B). Conversely, in cells pre-treated with the dual ET_A_R/ET_B_R antagonist macitentan, a FDA approved drug for the treatment of pulmonary arterial hypertension [27, 28], ET-1-induced ZEB1 and YAP/TAZ nuclear accumulation was inhibited (Fig. 1C, Additional file 3: Fig. S1A, B). Similarly, treatment with the selective ET_A_R antagonist BQ123, but not with the selective ET_B_R antagonist BQ788, impeded the ET-1-driven increase of ZEB1, YAP, and TAZ content in the nuclear compartment (Fig. 1D), suggesting the ET-1 ability to promote the parallel activation of ZEB1 and YAP/TAZ-triggered signals through the activation of ET_A_R in HG-SOC cells. In light of these results, a possible direct ZEB1/YAP interaction was investigated by performing co-immunoprecipitation (Co-IP) assay of endogenous ZEB1 and YAP in nuclear extracts from PD-HG-SOC cells. This analysis revealed a physical ZEB1/YAP interaction upon ET-1 stimulation that was disallowed by macitentan (Fig. 1E). Of note, proximity ligation assay (PLA) in PMOV10 and Kuramochi cells remarked the ability of ET-1 to favor the direct interaction between ZEB1 and YAP proteins as indicated by the increase of the fluorescent red dots upon ET-1 stimulation (Fig. 1F, Additional file 3: Fig. S1C). Altogether, these findings suggest the ET-1/ET_A_R capability to favour an interplay between ZEB1 and YAP signaling in HG-SOC cells.

### ET-1/ETAR axis promotes the engagement of YAP/AP-1/ZEB1 in a transcriptional nuclear complex

**Fig. 2:**
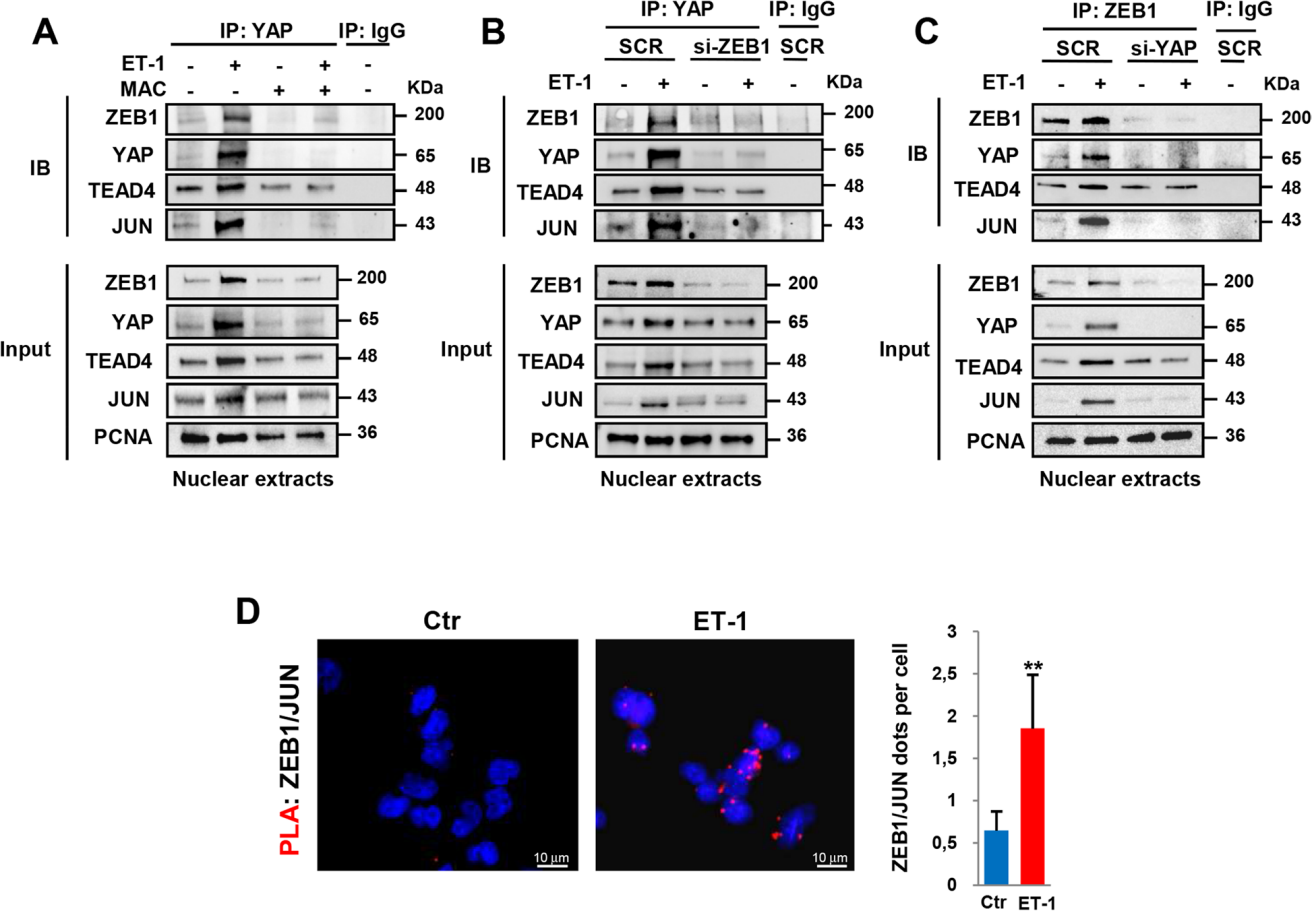
ET-1/ET_A_R axis promotes the engagement of the ZEB1/YAP/AP-1 transcriptional nuclear complex. **A** Nuclear extracts of PMOV10 cells stimulated for 6 h with ET-1 and/or with macitentan as indicated were IP for endogenous YAP using anti-YAP or anti-IgG as control Ab and IB using Abs recognizing the ZEB1, YAP, TEAD4, or c-JUN (JUN) proteins. PCNA was used as loading control. **B-C** Nuclear extracts of PMOV10 cells transfected with siRNA control (SCR) and si-ZEB1 (**B**) or si-YAP (**C**) for 72 h and stimulated for 6 h with ET-1 as indicated were IP using anti-IgG Ab control, anti-YAP Ab for endogenous YAP (**B**), and/or anti-ZEB1 Ab for endogenous ZEB1 (**C**), and IB as in *A*. **D** Representative images of PLA detection of protein complex containing ZEB1 and JUN (red signals) in PMOV10 cells stimulated or not with ET-1 for 6 h. DAPI staining (blue) highlights the nucleus (Magnification: 63x; scale bar: 10 μm). Right graph represents the quantification of the protein complex. Bars are means ± SD (*n* = 3; **p < 0.01)

Besides to act as a transcriptional repressor, ZEB1 can take action as a co-activator in DNA-binding transcriptional platforms with different partners, including YAP [21, 23–25]. To characterize the ZEB1/YAP transcriptional partners, we performed Co-IP assays by using nuclear extracts from PD-HG-SOC cells and cell line. In agreement with the dominant role of TEAD in forming DNA-binding platforms for YAP [18], we observed the presence of TEAD4, along with YAP and ZEB1 after IP of YAP or ZEB1 in cells stimulated with ET-1 (Fig. 2A-C, Additional file 3: Fig. S1D). Importantly, Co-IP experiments also revealed the ability of ET-1 to favour the recruitment in the ZEB1/YAP/TEAD4 complex of the AP-1 subunit JUN (Fig. 2A-C, Additional file 3: Fig. S1D) which has emerging as an important mediator in the YAP-induced downstream transcriptional effects [19, 21]. Conversely, the treatment with macitentan, as well as the depletion of ZEB1 and YAP, disabled the interaction of ZEB1/YAP with TEAD4 and JUN upon ET-1 (Fig. 2A-C, Additional file 3: Fig. S1D). Of note, in HG-SOC cells depleted for YAP, ET-1 was not able to upregulate JUN (Fig. 2C), suggesting a possible involvement of YAP in mediating the ET-1R-triggered AP-1 regulation. These results are in line with previous studies indicating that JUN is regulated by ET-1 signaling [29, 30] and is a transcriptional target of YAP [31]. Moreover, PLA revealed the capacity of ZEB1, beyond YAP, to directly interact with AP-1 in the nuclei of ET-1-stimulated PD-HG-SOC cells (Fig. 2D), thus indicating the existence of a transcriptional hub established by ET-1/ET_A_R signaling in HG-SOC cells and including ZEB1, YAP/TEAD and AP-1.

### YAP/AP-1/ZEB1 complex drives a feed-forward ET-1/ETAR signaling

**Fig. 3:**
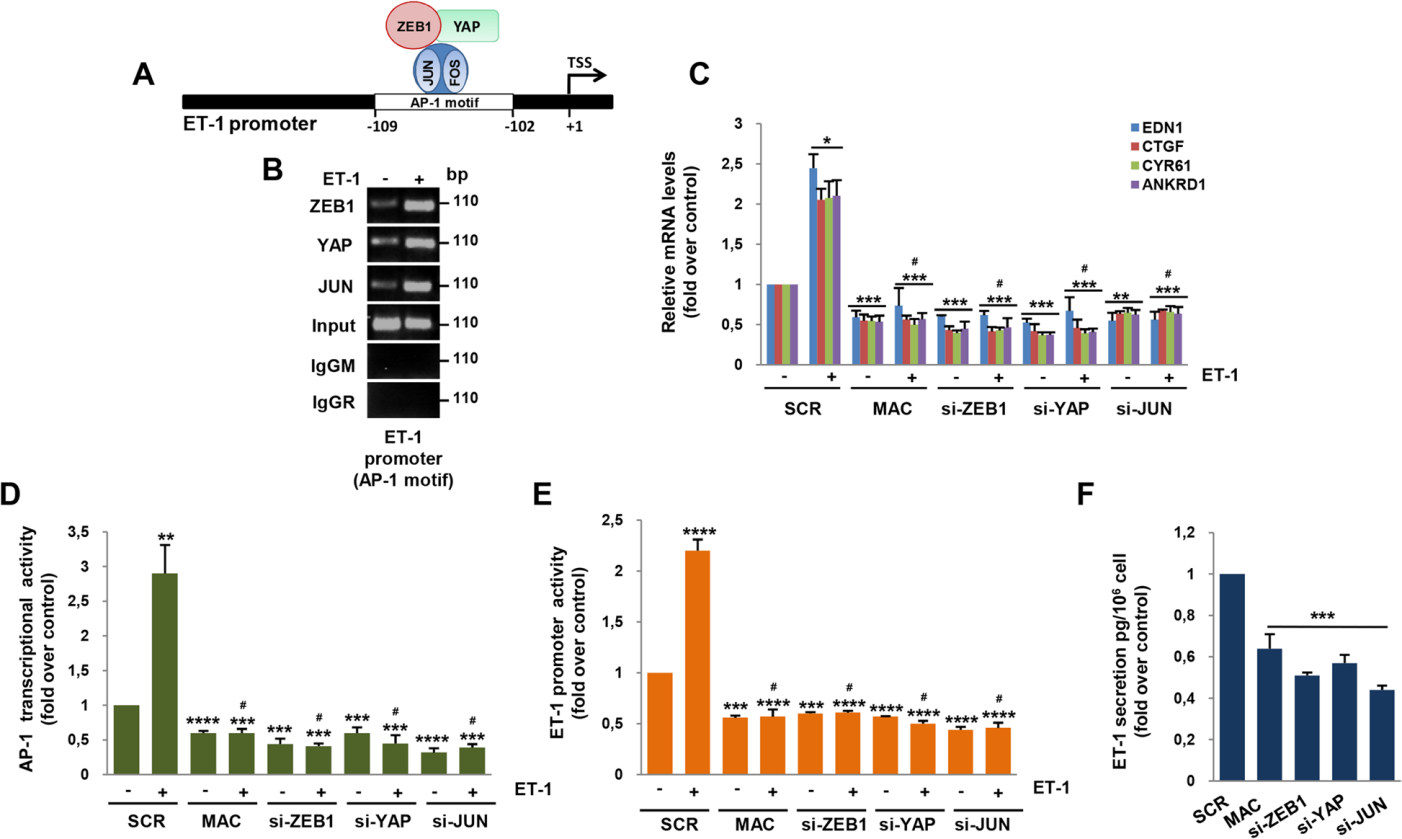
YAP/AP-1/ZEB1 complex drives a feed-forward ET-1/ET_A_R signaling. **A-B** The recruitment of ZEB1, YAP, and JUN proteins on the AP-1 motif of the ET-1 promoter region was analyzed by ChIP assay followed by PCR in PMOV10 cells stimulated or not with ET-1 for 6 h. **C** ET-1 (*EDN1*), *CTGF*, *CYR61* and *ANKRD1* gene expression in PMOV10 cells transfected with SCR, si-ZEB1, si-YAP, or si-JUN for 72 h and stimulated or not with ET-1 and/or MAC for 24 h was analyzed by q-RT-PCR and normalized to cyclophilin-A. Values are the means ± SD expressed as fold over control (n = 3; *p < 0.05; **p < 0.01; ***p < 0.001; #: *p* value was calculated vs. SCR + ET-1). **D** AP-1 transcriptional activity was analyzed in PMOV10 cells stimulated with ET-1 and/or treated with macitentan for 24 h and transfected with SCR, si-ZEB1, si-YAP, or si-JUN for 72 h together with the reporter plasmid containing a synthetic promoter target of AP-1 for 48 h. Values are the means ± SD expressed as fold over control (n = 3; **p < 0.01; ***p < 0.001; ****p < 0.0001; #: *p* value was calculated vs. SCR + ET-1). **E** ET-1 promoter activity was analyzed in PMOV10 cells stimulated as in *D* for 24 h and transfected with SCR, si-ZEB1, si-YAP, or si-JUN for 72 h together with a reporter plasmid containing the ET-1 promoter sequence for 48 h. Values are the means ± SD expressed as fold over control (n = 3; ***p < 0.001; ****p < 0.0001; #: *p* value was calculated vs. SCR + ET-1). **F** ET-1 release was evaluated by ELISA from conditioned media of PMOV10 cells transfected as in *C* or stimulated with macitentan for 72 h. Values are the means ± SD expressed as fold over control (n = 3; ***p < 0.001)

Given the pivotal role of AP-1 as a potent inducer of *EDN1* gene transcription [32, 33], and because ET-1 (*EDN1*) has been recently identified in a core set of common YAP/AP-1/ZEB1-activated target genes [21], we assessed whether ZEB1/YAP can form an active transcriptional complex on the AP-1 consensus site (GTGACTAA) of the ET-1 promoter (Fig. 3A). Chromatin immunoprecipitation (ChIP) assay revealed the co-occupancy of ZEB1, YAP, and JUN at this specific AP-1 binding site upon ET-1 stimulation (Fig. 3B), indicating the ability of AP-1 to act as a DNA anchor for ZEB1/YAP to mediate their control on the *EDN1* gene. Conversely, ZEB1, YAP, and JUN recruitment was not observed in a region of the ET-1 promoter lacking consensus sites for AP-1, nor for ZEB1 or TEAD4 (Additional file 3: Fig. S2A). The ability of ET-1 to guide a transcriptionally active YAP/AP-1/ZEB1 complex was further demonstrated by qRT-PCR experiments which provided evidence that in HG-SOC cells depleted for ZEB1, YAP, and JUN or treated with macitentan, ET-1 was not able to activate common YAP/AP-1/ZEB1 gene targets, including *EDN1*, as well as *CTGF*, *CYR61*, and *ANKRD1* genes (Fig. 3C, Additional file 3: Fig. S2B, C, S3A). In addition, we analyzed the regulatory function of the interplay between ET-1/ET_A_R and ZEB1/YAP axes on the AP-1 transcriptional activity by carrying out luciferase experiments with a synthetic promoter possessing repeated consensus sites for AP-1. In agreement with ChIP results, we noted an increase of the luciferase activity upon ET-1 stimulation that was inhibited by the depletion of both ZEB1 and YAP, similarly to the JUN silencing or macitentan treatment (Fig. 3D, Additional file 3: Fig. S3B), demonstrating the existence of an ET-1R-YAP/ZEB1 integrated network able to strength the AP-1 transcriptional activity. In light of the above results, we sought to elucidate the mechanism by which the YAP/AP-1/ZEB1 complex regulates *EDN1* gene expression. To this end, ET-1 transcription was firstly analyzed by performing luciferase experiments in HG-SOC cells transfected with a reporter construct containing the ET-1 promoter sequence. The activity of this construct was increased by ET-1 and this effect was lost in cells depleted for each transcriptional partner of the complex, as well as by blocking ET_A_R with macitentan (Fig. 3E, Additional file 3: Fig. S3C), indicating the involvement of this ET_A_R-activated YAP/AP-1/ZEB1 circuit in regulating ET-1 transcription. Intriguingly, the absence of consensus sites for ZEB1 on the ET-1 promoter sequence suggests that this factor was able to indirectly regulate ET-1 transcription by acting as a YAP/AP-1 co-activator. Most importantly, a significant reduction of the ET-1 protein released in the conditioned media from HG-SOC cells silenced for ZEB1, YAP, and JUN, or treated with macitentan was observed (Fig. 3F), suggesting a novel layer of signaling regulation activated by ET-1 to auto-reinforce its autocrine signals.

### YAP and AP-1 are involved in ET-1/ILK-induced transcriptional activation of ZEB1

**Fig. 4:**
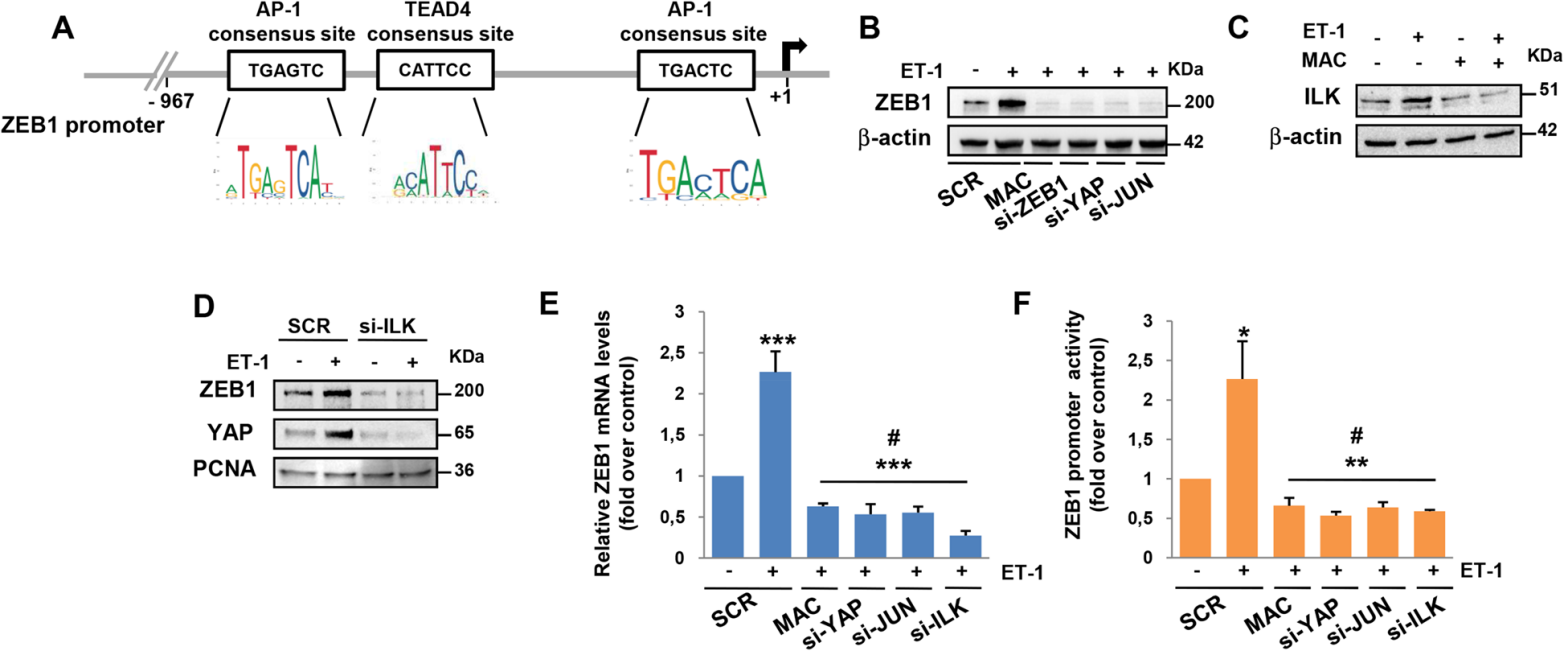
YAP and AP-1 are involved in ET-1/ILK-induced transcriptional activation of ZEB1. **A** Schematic representation of ZEB1 promoter reporter construct carrying the binding motifs of AP-1 and TEAD4 obtained from JASPAR. **B** Total extracts of PMOV10 cells transfected with SCR, si-ZEB1, si-YAP, or si-JUN for 72 h and stimulated with ET-1 and/or treated with macitentan for 48 h were IB with anti-ZEB1 Ab. β-actin was used as loading control. **C** Total extracts of PMOV10 cells stimulated with ET-1 and/or treated with macitentan for 30 min as indicated were IB with anti-ILK Ab. β-actin was used as loading control. **D** Nuclear extracts of PMOV10 cells transfected with SCR or si-ILK for 72 h and stimulated with ET-1 for 6 h were IB for ZEB1 or YAP. PCNA was used loading control. **E** ZEB1 gene expression in PMOV10 cells transfected for 72 h as in *B* or with si-ILK and stimulated as indicated for 24 h was analyzed by q-RT-PCR and normalized to cyclophilin-A. Values are the mean ± SD expressed as fold over control (*n* = 3; ***p < 0.001; #: *p* value was calculated vs. SCR + ET-1). **F** ZEB1 promoter activity was analyzed in PMOV10 cells stimulated for 24 h as indicated and transfected with SCR, si-ZEB1, si-YAP, si-JUN, or si-ILK for 72 h and together with a reporter plasmid for ZEB1 promoter for 48 h. Values are the means ± SD expressed as fold over control (n = 3; *p < 0.05; **p < 0.01; #: *p* value was calculated vs. SCR + ET-1)

Recent evidence identifies ZEB1 as a downstream target of the YAP/TEAD [34]. Because the analysis of ZEB1 promoter region 1000 bp upstream to the TSS revealed the presence of AP-1 consensus binding sites (Fig. 4A), we sought to define the role of both YAP and AP-1 in ET-1-dependent ZEB1 regulation. Importantly, Co-IP assay showed that the depletion of YAP determined a reduced nuclear expression of ZEB1 upon ET-1 stimulation (Fig. 2C). In light of these results, IB analyses of whole cell lysates from HG-SOC cells indicated that ET-1 was unable to increase the expression of ZEB1 in cells silenced for YAP, similarly to macitentan pre-treatment (Fig. 4B, Additional file 3: Fig. S4A, B). Moreover, these findings highlighted the role of JUN in regulating ZEB1 expression (Fig. 4B, Additional file 3: Fig. S4A, B). Given that previous evidence describing the ability of ILK to promote YAP nuclear translocation [35] and because we have previously pointed out that ILK is downstream to the ET-1 signaling to promote OC cell invasive behaviour [7, 9, 36], we then investigated the involvement of this kinase in mediating the ET-1-induced YAP activity. IB analyses confirmed the capacity of ET-1/ET_A_R axis to upregulate ILK that was curbed by macitentan pre-treatment (Fig. 4C, Additional file 3: Fig. S4C). Consequently, in HG-SOC cells depleted for ILK, ET-1 was not able to trigger the nuclear accumulation of YAP (Fig. 4D, Additional file 3: Fig. S2B, S4D-F). In a similar way, the depletion of ILK affected the ET-1-driven ZEB1 nuclear enrichment (Fig. 4D, Additional file 3: S2B, Fig. S4D-F), demonstrating an essential role of this kinase in ET-1-induced YAP/ZEB1 signaling. Next, we evaluated whether ILK, YAP, and AP-1 modulated ZEB1 at transcriptional level. In this regard, a reduction of ZEB1 mRNA expression was observed after the depletion of these factors upon ET-1 stimulation (Fig. 4E). Furthermore, luciferase experiments demonstrated the ability of ET-1 to enhance ZEB1 transcription only in cells expressing ILK, YAP, and JUN (Fig. 4F). Collectively, these results reveal that ILK, YAP, and AP-1 signaling pathways converge in mediating the ET-1-induced transcription of ZEB1, underlying a novel regulatory network between ET-1, YAP, and ZEB1 as critical determinants of HG-SOC progression.

### ZEB1/YAP signaling is involved in ET-1R/ILK-induced ovarian cancer aggressiveness

**Fig. 5:**
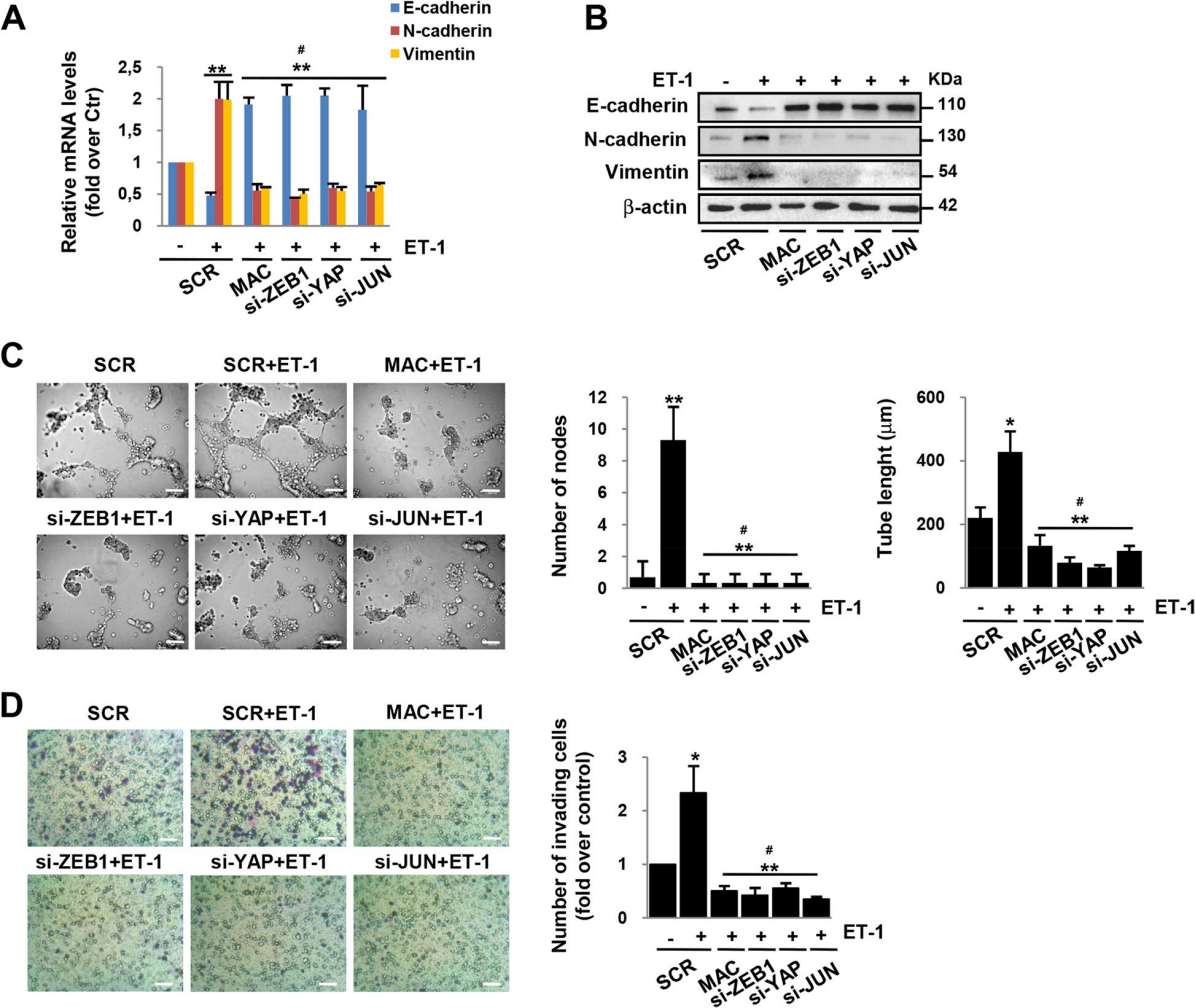
YAP/AP-1/ZEB1 complex is involved in ET-1-induced EMT, cellular plasticity, and cell invasion. **A** E-cadherin, N-cadherin and Vimentin gene expression in OVCAR-3 cells transfected with SCR, si-ZEB1, si-YAP, or si-JUN for 72 h and stimulated with ET-1 and/or treated with macitentan for 24 h as indicated was analyzed by q-RT-PCR and normalized to cyclophilin-A. Values are the means ± SD expressed as fold over control (n = 3; **p < 0.01; #: *p* value was calculated vs. SCR + ET-1). **B** Total extracts of OVCAR-3 cells transfected for 72 h and stimulated for 48 h as in *A* were IB with anti-E-cadherin, anti-N-cadherin or anti-Vimentin Abs. β-actin was used as loading control. **C** Vasculogenic mimicry assay with OVCAR-3 cells transfected for 48 h and overnight stimulated as in *A* (Magnification: 20x; scale bar: 100 μm). Right graphs represent the quantification of the number of nodes and the tube length. Columns show the mean ± SD (n = 3; *p < 0.05; **p < 0.01; #: *p* value was calculated vs. SCR + ET-1). **D** Transwell chemoinvasion assay with OVCAR-3 cells transfected as in *A* and overnight allowed to invade in presence of ET-1 and/or macitentan. Images represent the crystal violet-stained invasive cells (Magnification: 20x; scale bar: 100 μm). Right graph represents the number of invading cells. Values are the means ± SD expressed as fold over control (n = 3; *p < 0.05; **p < 0.01; #: *p* value was calculated vs. SCR + ET-1)

Given the important role of ET-1/ET_A_R axis to favor EMT and HG-SOC progression through different signaling, including ILK [7, 9], and the key role of ZEB1 in EMT process [5, 11], the contribute of ZEB1/YAP signaling in mediating ET-1R/ILK-driven EMT was assessed. Interestingly, ET-1-induced expression of mesenchymal markers, N-cadherin and vimentin, and reduced expression of the epithelial marker E-cadherin were inhibited, at both mRNA and protein levels, upon the depletion of ILK, ZEB1, YAP, and JUN or following macitentan treatment (Fig. 5A, B, Additional file 3: Fig. S4D, S5A-C, S6A, B). Moreover, we evaluated the involvement of the ZEB1/YAP interplay in the ET-1 capacity to regulate cellular plasticity, as the ability of aggressive cells to form vascular-like structures in the vasculogenic mimicry assay [37]. ET-1-stimulated HG-SOC cells were able to organize themselves into vascular-like structures forming a greater number of nodes and longer tubes than unstimulated cells (Fig. 5C, Additional file 3: Fig. S6C). Interestingly, the depletion of ZEB1, YAP, JUN, or ILK significantly disrupted the formation of these structures, similarly to macitentan (Fig. 5C, Additional file 3: Fig. S6C), indicating an important role of the ZEB1/YAP signaling in phenotypic plasticity induced by ET-1. Since invasive cell behaviour is a metastatic trait essential to cancer dissemination, we performed transwell cell invasion assays to further elucidate how the integration of ET-1R signaling with the YAP/ZEB1 axis may impinge in the acquisition of this aggressive feature. These experiments revealed an enhanced ability of HG-SOC cells to invade through a matrix mimicking the basal membrane in response to the chemo-attractant effect of ET-1 (Fig. 5D, Additional file 3: Fig. S5D, S6D). Of note, cells depleted for ZEB1, YAP, JUN, and ILK or treated with macitentan, were less prone to invade in response to ET-1 (Fig. 5D, Additional file 3: Fig. S5D, S6D), indicating a key functional output of the ILK/YAP/AP-1/ZEB1 cooperation in mediating ET-1-driven EMT, cellular plasticity and invasion in HG-SOC cells.

### Targeting ETAR with macitentan impairs metastatic spread by interfering with the ZEB1/YAP signaling

**Fig. 6:**
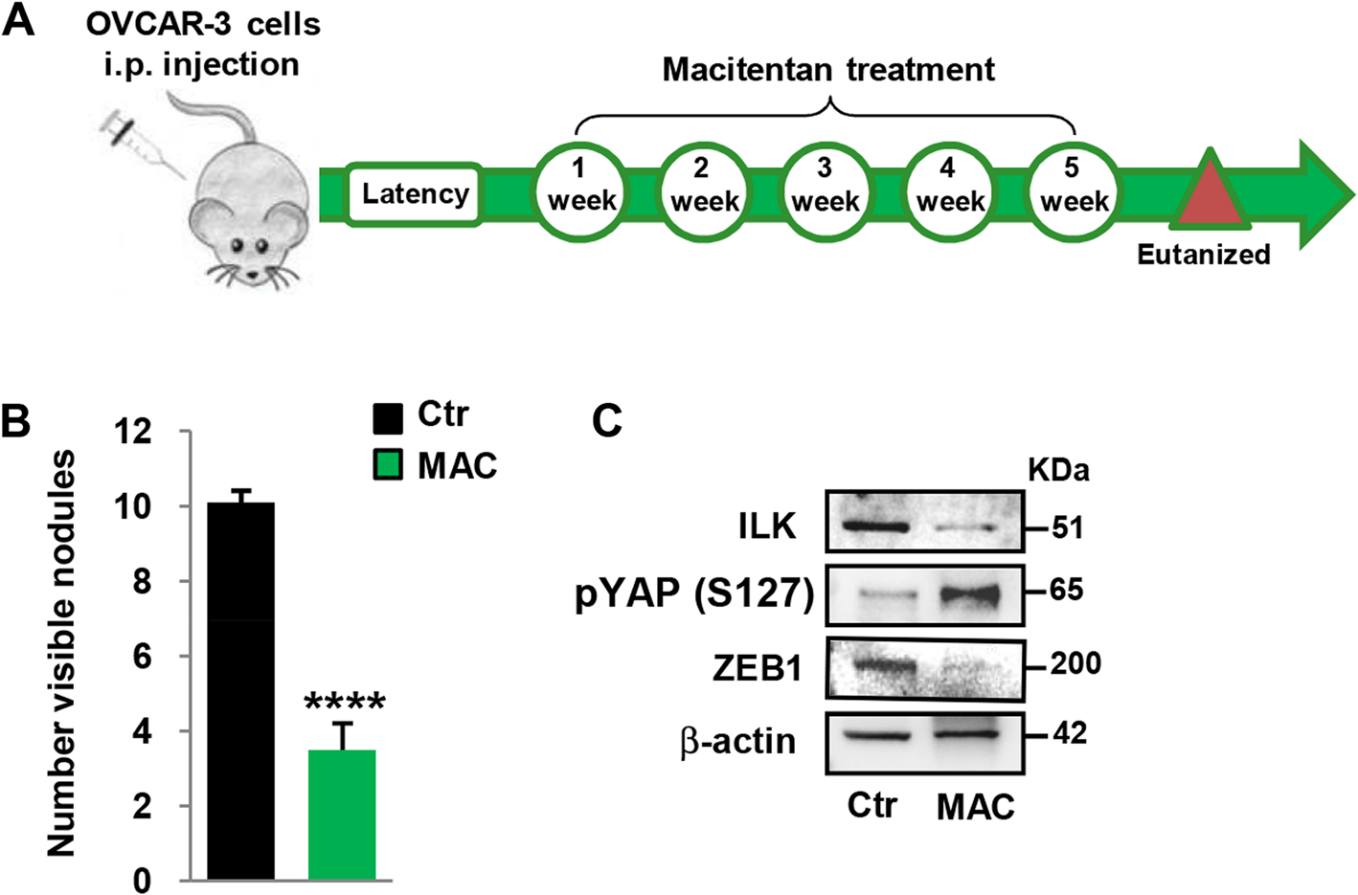
Targeting ET_A_R with macitentan impairs metastatic formation in vivo by interfering with the ZEB1/YAP signaling. **A**, **B** Female nude mice were intraperitoneally (i.p.) injected with OVCAR-3 cells and, after 1 week of latency, were treated with vehicle (Ctr) or MAC (30 mg/kg oral daily) for 5 weeks. Graph (**B**) represents the number of visible metastasis. Values are the means ± SD (****p < 0.0001). **C** IB analysis for ZEB1, pYAP (S127), and ILK in total extracts from i.p. nodules of OVCAR-3 xenografts treated as in *A*. β-actin was used as loading control

To evaluate the impact of ET-1-YAP/ZEB1 network on metastatic progression in vivo, we generated orthotopic HG-SOC xenografts by intraperitoneally injecting OVCAR-3 cells in nude mice and treating them with macitentan or vehicle for 5 weeks (Fig. 6A). In agreement with the critical role of ET-1/ET-1R signaling in HG-SOC metastatic spreading [9], we observed a reduced number of intraperitoneal metastatic nodules in the group of mice in which ET-1R signaling was blocked by macitentan (Fig. 6B), which was associated with a well-tolerated toxicity profile (no weight loss in the treated mice). Importantly, IB analysis of lysates from intraperitoneal metastatic nodules revealed that ET-1R blockade by macitentan concomitantly inhibited ILK/ZEB1 expression and YAP activity (Fig. 6C). Altogether, these in vivo findings strength the importance of an ET-1R-activated ZEB1/YAP circuit ​in metastasis formation, thus suggesting the hampering of this circuit by ET-1R antagonist as a potential therapeutic strategy for the treatment of metastatic HG-SOC.

### ETAR/ILK/YAP/AP-1/ZEB1 gene signature correlates with a poor prognosis in ovarian cancer patients

**Fig. 7:**
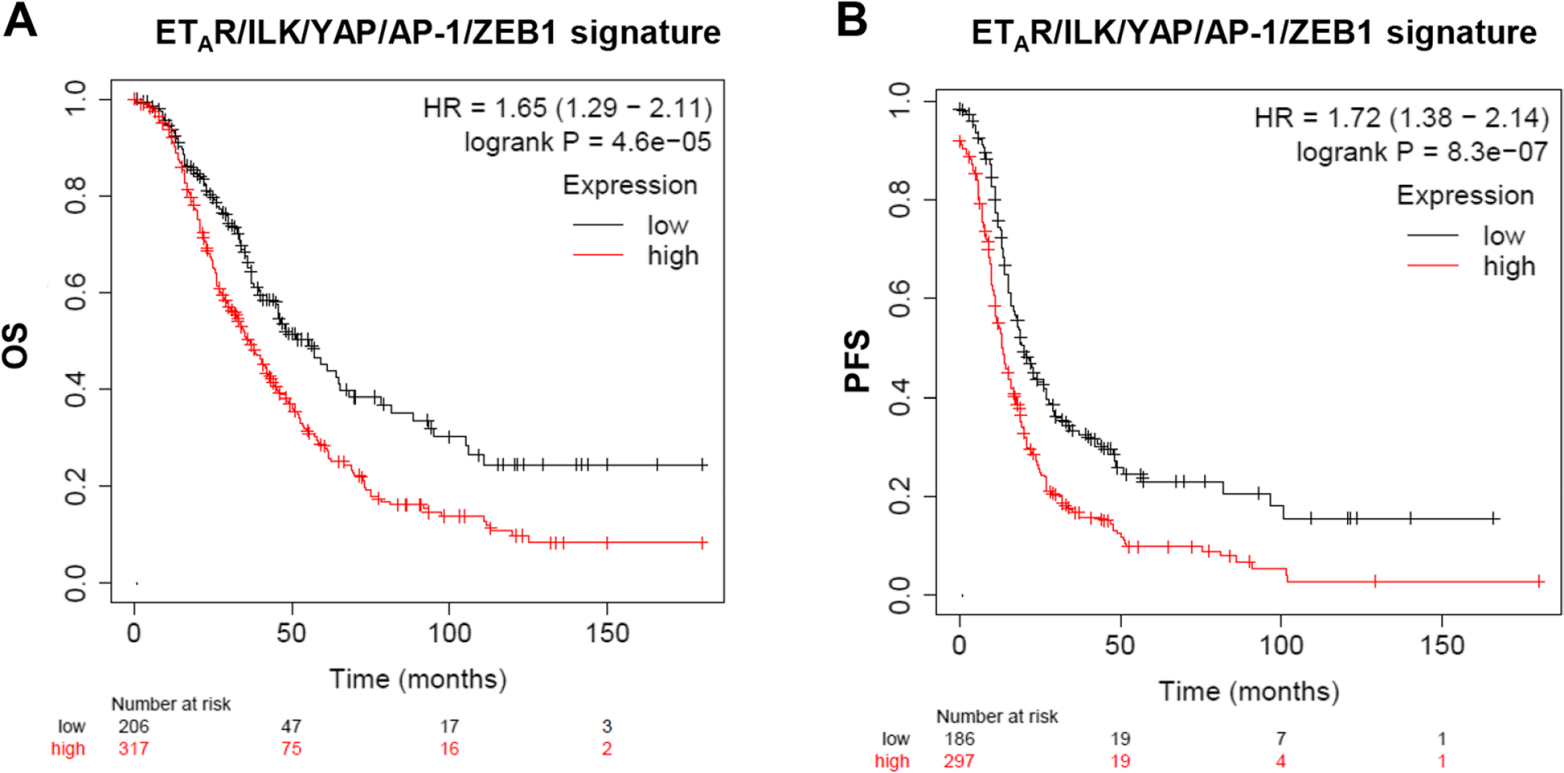
High expression of the ET_A_R/ILK/YAP/AP-1/ZEB1 gene signature correlates with a poor prognosis in ovarian cancer patients. **A** Kaplan–Meier (KM) curves of overall survival (OS) generated by the analysis of serous OC patients from KM-plotter database (kmplot.com) grouped in high (317 patients, red line) and low (206 patients, black line) expression levels of the ET_A_R/ILK/YAP/AP-1/ZEB1 gene signature (*p* = 4.6e-05). **B** KM curves of progression-free survival (PFS) generated by the analysis of serous OC patients from KM-plotter database grouped in high (297 patients, red line) and low (186 patients, black line) expression levels of the ET_A_R/ILK/YAP/AP-1/ZEB1 gene signature (*p* = 8.3e-07)

To evaluate the prognostic relevance of the functional integration of ET-1/ET_A_R/ILK and YAP/AP-1/ZEB1 axes we analyzed a cohort of serous OC patients from KM plotter [26]. We dichotomized patients in high or low mRNA levels of ET_A_R, ILK, YAP, AP-1, or ZEB1 alone or in combination and found that patients who expressed high mRNA levels of a broader integrated ET_A_R/ILK/YAP/AP-1/ZEB1 signature exhibited worsening prognosis in terms of overall survival (OS) (hazard ratio (HR) = 1.65 [95% CI: 1.29–2.11], *p* = 4.6e-05) (Fig. 7A), as well as progression-free survival (PFS) (HR = 1.72 [95% CI: 1.38–2.14], *p* = 8.3e-07) (Fig. 7B), compared to patients expressing lower mRNA levels of these genes. Overall, our study provides important evidence of the clinical value of the ET_A_R/ILK/YAP/AP-1/ZEB1 gene signature in predicting the clinical outcome of OC patients.

## Discussion

**Fig. 8:**
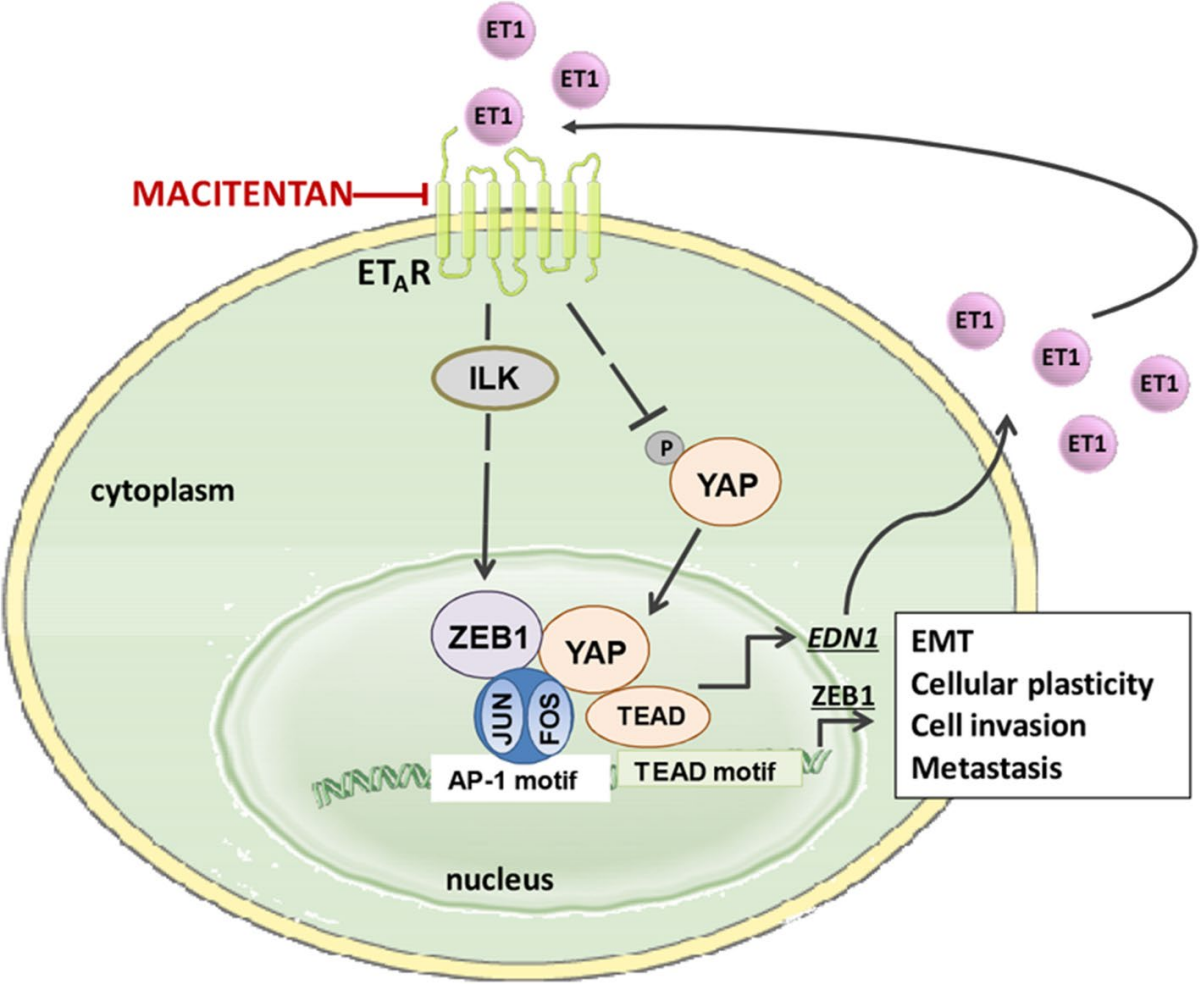
A schematic diagram illustrates the potential mechanism by which ET_A_R drives YAP/ZEB1 signaling network to promote metastatic traits in HG-SOC cells. ET_A_R activation by ET-1 increases ILK expression which, in turn, promotes the YAP/ZEB1 nuclear accumulation. In the nucleus, YAP and ZEB1 are engaged in an active transcriptional complex with AP-1 that favors the ZEB1 and ET-1 production, thereby creating a feed-forward loop that sustains a persistent ET-1/ET_A_R signaling activity. YAP/ZEB1 interplay is involved in ET-1R/ILK-induced EMT, cellular plasticity, and cell invasion. ET_A_R blockade with macitentan, curbing the ZEB1/YAP transcriptional activity, prevents HG-SOC metastatic progression. Part of the figure is drawn using pictures from Servier Medical Art (https://smart.servier.com), licensed under a Creative Commons Attribution 3.0 Unported License (https://creativecommons.org/licenses/by/3.0)

Understanding the molecular mechanisms of metastatic progression may pave the way for new therapeutic strategies for the improvement of the clinical outcome of HG-SOC patients. The metastatic process is regulated by the complex changes in EMT-associated cellular phenotype, which are the results of a fine-tuned balance and cooperation of regulatory networks involving EMT-TF [4, 5]. In an attempt to decipher the complexity of the transcriptional framework underlying EMT, here we unveil a functional integration between ZEB1 and YAP signaling in mediating the ET-1/ET_A_R-triggered aggressive outputs in HG-SOC. We provide evidence that ET_A_R activation can engender a highly multi-factorial transcriptional program through the formation of a DNA-binding platform including ZEB1, YAP, and AP-1. Moreover, we demonstrate that ET-1-induced ILK sustains the activation of YAP/ZEB1 signaling conferring an advantage to the ET-1-driven EMT, cellular plasticity, and invasiveness. Blocking this circuit, by using the ET-1R antagonist macitentan, curbs metastasis formation in HG-SOC xenografts (Fig. 8).

ZEB1 and YAP represent two important routes for cancer development towards metastasis which are hyperactivated in different type of tumors, including OC [10, 11, 14–16]. Our recent discoveries in HG-SOC reveal the ET-1/ET_A_R axis ability to upregulate ZEB1 expression [10] and, in parallel, to induce YAP nuclear translocation [14, 15]. In this study, we demonstrate that ET_A_R activation determines the concomitant YAP/ZEB1 nuclear accumulation that endorsed their direct physical interaction. Of note, here we show an highly cooperative system in which ZEB1 and YAP act as co-activators in a transcriptional complex with AP-1 subunit JUN to mediate the dynamics of the ET-1 signaling-induced EMT. The cross-talk of ZEB1, YAP, and AP-1 pathways has been reported in a study performed in normal murine mammary gland as major signaling hubs in EMT [38]. Moreover, a functional interaction of these factors has been described in melanoma [39] and in breast cancer [21] cells. Interestingly, here we uncover a more intricate picture identifying ET-1R as a specific actionable target able to activate the YAP/AP-1/ZEB1 network. The consequent enhanced levels of *EDN1*, as a specific target gene, can elicit a self-amplifying circuit potentiating the ET-1R-driven adverse outcomes. This observation is supported by previous studies reporting that OC cells release ET-1 in their conditioned media to a concentration that is within the biologically effective range for this peptide, to ensure the ET-1 binding to the ET-1R [40, 41]. These findings imply that ET-1 sustains tumor growth and progression through an autocrine feed-forward loop that may represent a magnifying persistent mechanism in OC cells. Furthermore, we underscore the existence of a novel layer of inter-pathway regulation in which important EMT-related cues converge to modulate the abundance of ZEB1, the central hub of cellular plasticity during metastatic cascade [11]. Indeed, our results indicate the function of ILK, able to control ZEB1 expression [42], in mediating the ET-1/ET_A_R axis-dependent regulation of ZEB1.

In line with an evidence in colorectal cancer identifying ZEB1 as a YAP/TEAD target gene [34], in this study we reveal the ability of ET-1 to activate both YAP and AP-1 pathways which, in turn, are involved in mediating the ET-1/ET_A_R axis transcriptional regulation of ZEB1. On the other hand, ZEB1, as a bivalent activator of ET-1R signaling able to sustain the release of ET-1, as well as the ET_A_R expression [10], can impinge on the ET-1-triggered activity of both YAP and AP-1 signaling. Our results add greater evidence to previous discoveries highlighting the capacity of YAP to impact on AP-1 signaling [31, 43], attesting that YAP inactivation may impair the ET-1-induced AP-1 function, thus describing the existence of a reciprocal network that integrates ET-1R/ILK and ZEB1/YAP axes in a regulatory circuit to promote the acquisition of metastatic traits.

Analyses of human serous ovarian cancers sustain the translational and clinical relevance of our findings. Particularly, the co-expression of ET_A_R/ILK/YAP/AP-1/ZEB1 has a strong predictive potential of poor overall and relapse free survival, which suggests the worse outcomes generated by the integration between this transcriptional machinery for these patients still suffering from limited treatment option.

ZEB1 and YAP represent well-known vulnerabilities that could be exploited for the treatment of metastatic tumors. Due to the difficulties to directly target metastatic drivers as ZEB1 and YAP, challenging approaches may be based on the interfering with upstream actionable target affecting their intracellular activity. In this regard, the treatment with macitentan, able to exert anti-metastatic effect by disallowing the ET-1R/ILK/YAP/ZEB1 circuit, may represent a valuable therapeutic option in HG-SOC. The pharmacologic advantage of the dual ET_A_R/ET_B_R-antagonist is to target not only OC cells expressing ET_A_R, hampering the ZEB1/YAP interplay, but also to interfere with tumor microenvironment (TME) elements, such as cancer-associated fibroblasts, blood, lymphatic, and immune cells, which mainly expressed ET_B_R [9, 15, 44], representing a feasible approach which may target ET-1R-driven regulatory circuits in the HG-SOC ecosystem. Future research along these findings may disclose further layers of complexity in the effect of ET-1 signaling on the interplay between HG-SOC cells and TME.

## Conclusions

Overall, our findings reveal the ET-1R-driven transcriptional circuits controlling HG-SOC metastatic competence. Our results underline the cooperation of ZEB1 and YAP signaling downstream of ET-1R/ILK pathway to sustain a complex transcriptional program driving EMT and metastatic formation in HG-SOC preclinical models. These findings provide new insight into the therapeutic potential of ET-1R antagonists for targeting difficult intracellular protein–protein interaction, as YAP/AP-1/ZEB1 network, laying the groundwork for novel options of pharmacological interventions in metastatic HG-SOC.

## Supplementary Information

**Additional file 1.** 

**Additional file 2.**

**Additional file 3. **

**Additional file 4. **

## Data Availability

All data generated or analyzed during this study are included in this published article and its supplementary information files.

## Abbreviations

HG-SOC: High-grade serous ovarian carcinoma
OC: Ovarian carcinoma
ET-1: Endothelin-1
GPGR: G-protein coupled receptors
ET_A_R: Endothelin A receptor
ET_B_R: Endothelin B receptor
ET-1R: Endothelin-1 receptors
EMT: Epithelial-to-mesenchymal transition
TF: Transcription factors
ZEB1: Zinc finger E-box binding homeobox 1
YAP: Yes-associated protein
TAZ: Transcriptional coactivator with PDZ-binding motif
TEAD: Transcriptional coactivator enhanced associate domain
AP-1: Activator protein-1
ILK: Integrin-linked kinase
siRNA: Small interfering RNA
Ab: Antibody
IB: Immunoblotting
IP: Immunoprecipitation
Co-IP: Co-immunoprecipitation
PLA: Proximity ligation assay
ChIP: Chromatin immunoprecipitation
KM: Kaplan–Meier
OS: Overall survival
PFS: Progression free survival
HR: Hazard ratio
TME: Tumor microenvironment
