# Supplementary material for: Functional interaction between endothelin-1 and ZEB1/YAP signaling regulates cellular plasticity and metastasis in high-grade serous ovarian cancer
Source: J Exp Clin Cancer Res. 2022 Apr 28;41:157. doi: 10.1186/s13046-022-02317-1 (PMC9047299; doi:10.1186/s13046-022-02317-1)
Supplement: Supplementary file 1 — Additional file 1. [file 13046_2022_2317_MOESM1_ESM.doc]

**Additional file 2:** Table S2.

**Table S2.** Primer sequences used in this study.

| **Primer sequences used for qRT-PCR experiments** | | |
| --- | --- | --- |
| **Gene** | **Forward primer (5ˈ to 3ˈ)** | **Reverse primer (5ˈ to 3ˈ)** |
| *EDN1*  CYR61  CTGF  ANKRD1  E-cadherin  N-cadherin  Vimentin  ZEB1  Cyclophilin-A | CCAAGAGAGCCTTGGAGAAT  CAGGACTGTGAAGATGCGGT  AGGAGTGGGTGTGTGACGA  AGTAGAGGAACTGGTCACTGG  ACACCATCCTCAGCCAAGATCC  GGTGGAGGAGAAGAAGACCAG  TTTGAAGAAACTCCACGAAGAGGA  GCAGTCCAAGAACCACCCTT  TTCATCTGCACTGCCAAGAC | TGTCTTCAGCCCTGAGTTCTT  GCCTGTAGAAGGGAAACGCT  CCAGGCAGTTGGCTCTAATC  TGGGCTAGAAGTGTCTTCAGA  GTGGTGGGATTGAAGATCGGAG  GGCATCAGGCTCCACAGT  CCACATCGATTTGGACATGCT  GGGCGGTGTAGAATCAGAGT  TCGAGTTGTCCACAGTCAGC |
| **Primer sequences used for ChIP experiments** | | |
| **ET-1 promoter region** | **Forward primer (5ˈ to 3ˈ)** | **Reverse primer (5ˈ to 3ˈ)** |
| AP-1 site  Negative control | AAGTTAGCAGTGATTTCCTTTCG  GCAAATTCAGGATTCAAAGAGAC | TCTCTGCCGGCTTTTTATATTGA  TCCCGTGAATATGAATTGGAAAC |
