# Supplementary material for: Functional interaction between endothelin-1 and ZEB1/YAP signaling regulates cellular plasticity and metastasis in high-grade serous ovarian cancer
Source: J Exp Clin Cancer Res. 2022 Apr 28;41:157. doi: 10.1186/s13046-022-02317-1 (PMC9047299; doi:10.1186/s13046-022-02317-1)
Supplement: Supplementary file 2 — Additional file 2. [file 13046_2022_2317_MOESM2_ESM.doc]

**Additional file 1:** Table S1.

**Table S1.** Antibodies used in this study for immunoblotting analyses.

| **Antigen** | **Dilution** | **Manufacturer** |
| --- | --- | --- |
| PCNA (F-2) sc-25280  Tubulin (DM1A) sc-32293  ZEB1 (H-3) sc-515797  pYAP (S127) (D9W2I) cat. #13008  YAP (1A12) cat. #12395  pTAZ (S89) (E1X9C) cat. #59971  TAZ (V386) cat. #4883S  TEAD4 (5H3) ab58310  c-JUN (60A8) cat. #9165S  ILK cat. #GTX101691  E-cadherin (36) cat. #610181  N-cadherin (32) cat. #610921  Vimentin (D21H3) cat. #5741  β-actin (AC-15) cat. #1978 | 1:200  1:2000  1:1000  1:1000  1:1000  1:1000  1:1000  1:500  1:1000  1:500  1:500  1:500  1:1000  1:5000 | Santa Cruz Biotechnology  Santa Cruz Biotechnology  Santa Cruz Biotechnology  Cell Signaling Technology  Cell Signaling Technology  Cell Signaling Technology  Cell Signaling Technology  Abcam  Cell Signaling Technology  Genetex  BD Biosciences  BD Biosciences  Cell Signaling Technology  Sigma-Aldrich |
