# Supplementary material for: Functional interaction between endothelin-1 and ZEB1/YAP signaling regulates cellular plasticity and metastasis in high-grade serous ovarian cancer
Source: J Exp Clin Cancer Res. 2022 Apr 28;41:157. doi: 10.1186/s13046-022-02317-1 (PMC9047299; doi:10.1186/s13046-022-02317-1)
Supplement: Supplementary file 4 — Additional file 4. [file 13046_2022_2317_MOESM4_ESM.pdf]

**Additional file 3:** Supplementary Figures.

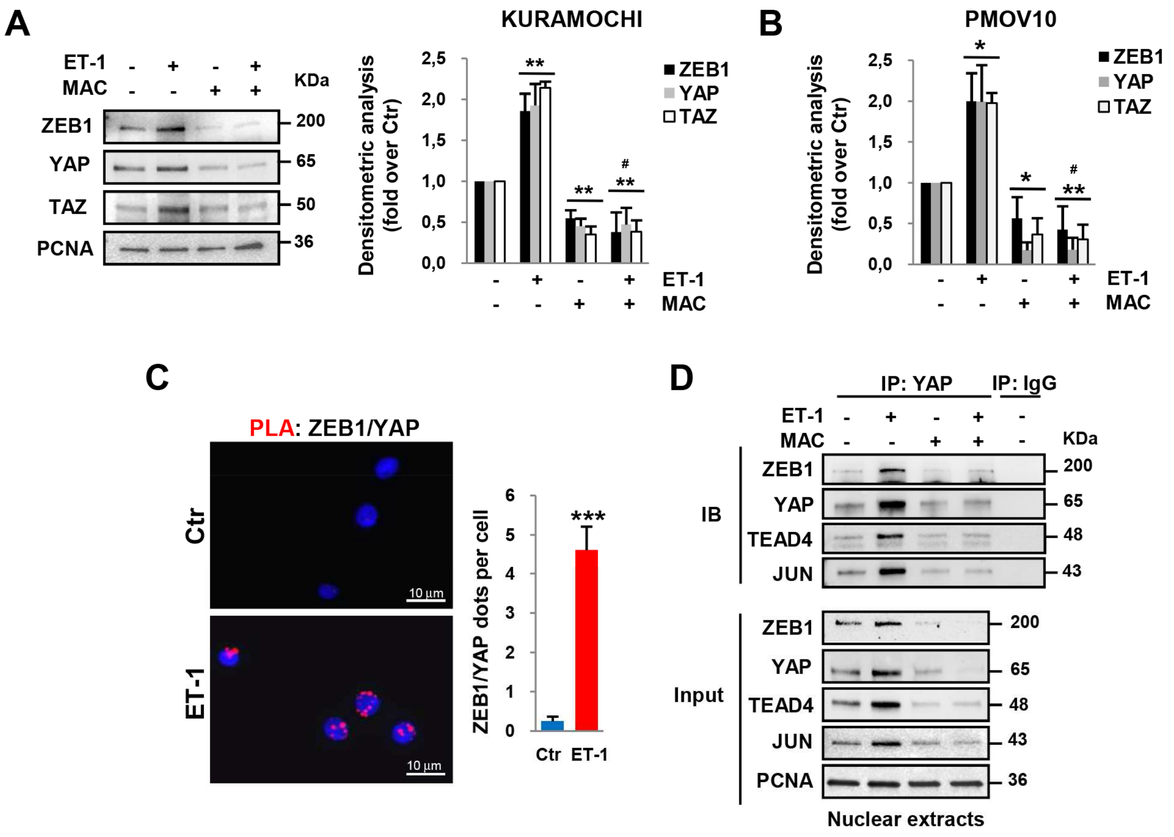


**Supplementary Figure S1.** ET-1/ET$_A$R axis promotes the engagement of the YAP/AP-1/ZEB1 transcriptional nuclear complex. **A** Nuclear extracts of Kuramochi cells stimulated for 6 hours (h) with ET-1 (100nM) and/or with macitentan (MAC, 1µM), a dual ET-1 receptor antagonist, were immunoblotted (IB) for ZEB1, YAP, and TAZ. Right graph represents the densitometric analysis of ZEB1, YAP, and TAZ protein expression normalized to PCNA. Values are the mean ±SD expressed as fold induction (n=3; ***p*<0.01; #: *p* value was calculated vs. ET-1). **B** Densitometric analysis of ZEB1, YAP, and TAZ protein expression normalized to PCNA of IB with nuclear extracts of PMOV10 cells treated as in *A*. Values are the mean ±SD expressed as fold induction (n=3; **p*<0.05; ***p*<0.01; #: *p* value was calculated vs. ET-1). **C** Representative images of proximity ligation assay (PLA) detection of direct protein-protein interaction between ZEB1 and YAP (red signals) in Kuramochi cells stimulated or not with ET-1 for 6 h. DAPI staining (blue)

highlights the nucleus (Magnification: 63x; scale bar: 10 μm). Right graph represents the quantification of the ZEB1/YAP protein interaction. Bars are means ±SD (n = 3; ***$p$ <0.001). **D** Nuclear extracts of OVCAR-3 cells stimulated for 6 h with ET-1 and/or with MAC as indicated were immunoprecipitated (IP) for endogenous YAP using anti-YAP antibody (Ab) or anti-IgG as control Ab and immunoblotted (IB) using Abs recognizing the ZEB1, YAP, TEAD4, or c-JUN (JUN) proteins. PCNA was used as loading control.

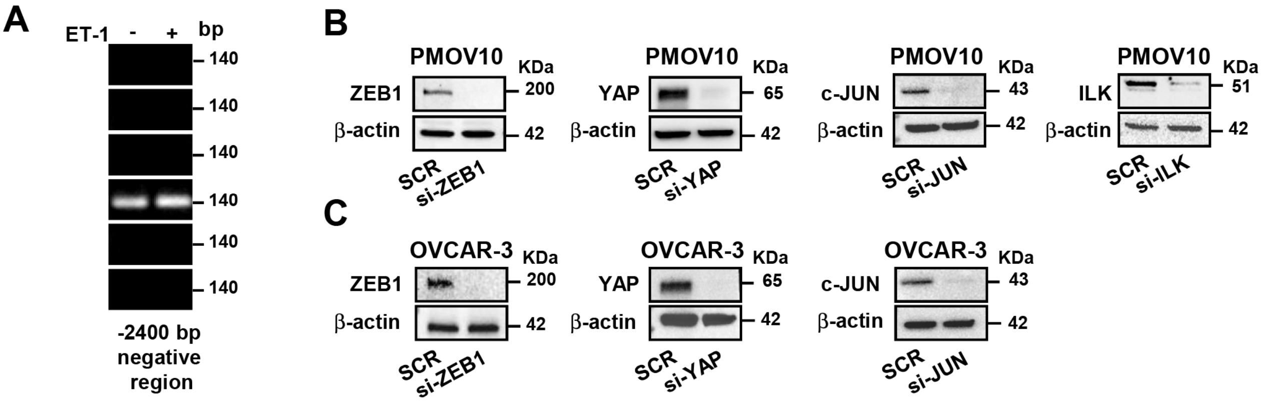


**Supplementary Figure S2.** ChIP assay and siRNA transfection controls. **A** The recruitment of YAP/AP-1/ZEB1 complex on a region -2400 bp upstream the ET-1 TSS site (negative control for non-specific enrichment) was analyzed by ChIP assay followed by PCR in PMOV10 cells stimulated or not with ET-1 for 6 h. **B-C** IB analyses for the indicated proteins in total extracts of PMOV10 (**B**) and OVCAR-3 (**C**) cells transfected for 72 h with SCR, si-ZEB1, si-YAP, si-JUN, or si-ILK. β-actin was used as loading control.

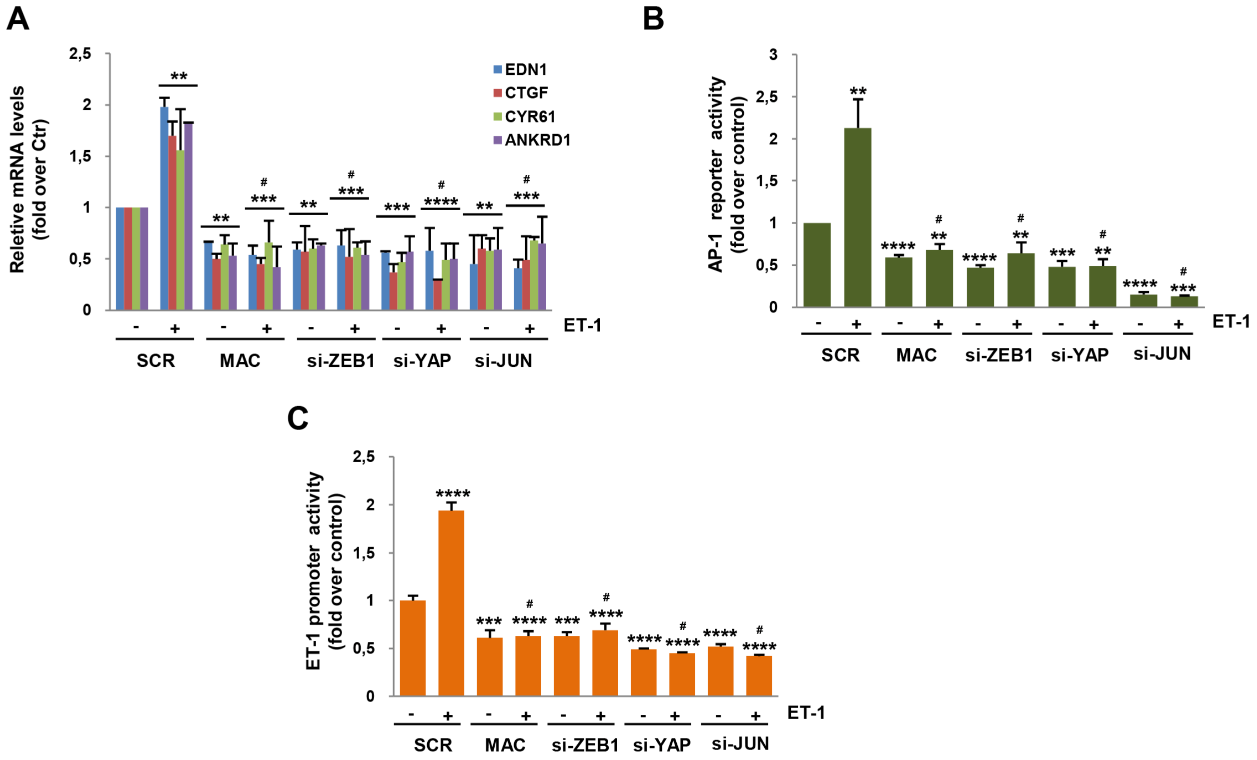


**Supplementary Figure S3.** YAP/AP-1/ZEB1 complex drives a feed-forward ET-1/ET$_A$R signaling. **A** ET-1 (*EDN1*), *CTGF*, *CYR61*, and *ANKRD1* gene expression in OVCAR-3 cells transfected with SCR, si-ZEB1, si-YAP, or si-JUN for 72 h and stimulated or not with ET-1 and/or macitentan for 24 h was analyzed by q-RT-PCR and normalized to cyclophilin-A. Values are the means ± SD expressed as fold over control (n = 3; ***p* < 0.01; ****p* < 0.001; *****p* < 0.0001; #: *p* value was calculated vs. SCR+ET-1). **B** AP-1 reporter activity was analyzed in OVCAR-3 cells stimulated with ET-1 and/or treated with macitentan for 24 h as indicated and transfected for 48 h with SCR, si-ZEB1, si-YAP, or si-JUN together with a reporter plasmid containing a synthetic AP-1 target promoter. Values are the means ± SD expressed as fold over control (n = 3; ***p* < 0.01; ****p* < 0.001; *****p* < 0.0001; #: *p* value was calculated vs. SCR+ET-1). **C** ET-1 promoter activity was analyzed in OVCAR-3 cells stimulated as in *B* as indicated and transfected for 48 h with SCR, si-ZEB1, si-YAP, or si-JUN together with a reporter plasmid containing the ET-1 promoter sequence. Values are the means ± SD expressed as fold over control (n = 3; ****p* < 0.001; *****p* < 0.0001; #: *p* value was calculated vs. SCR+ET-1).

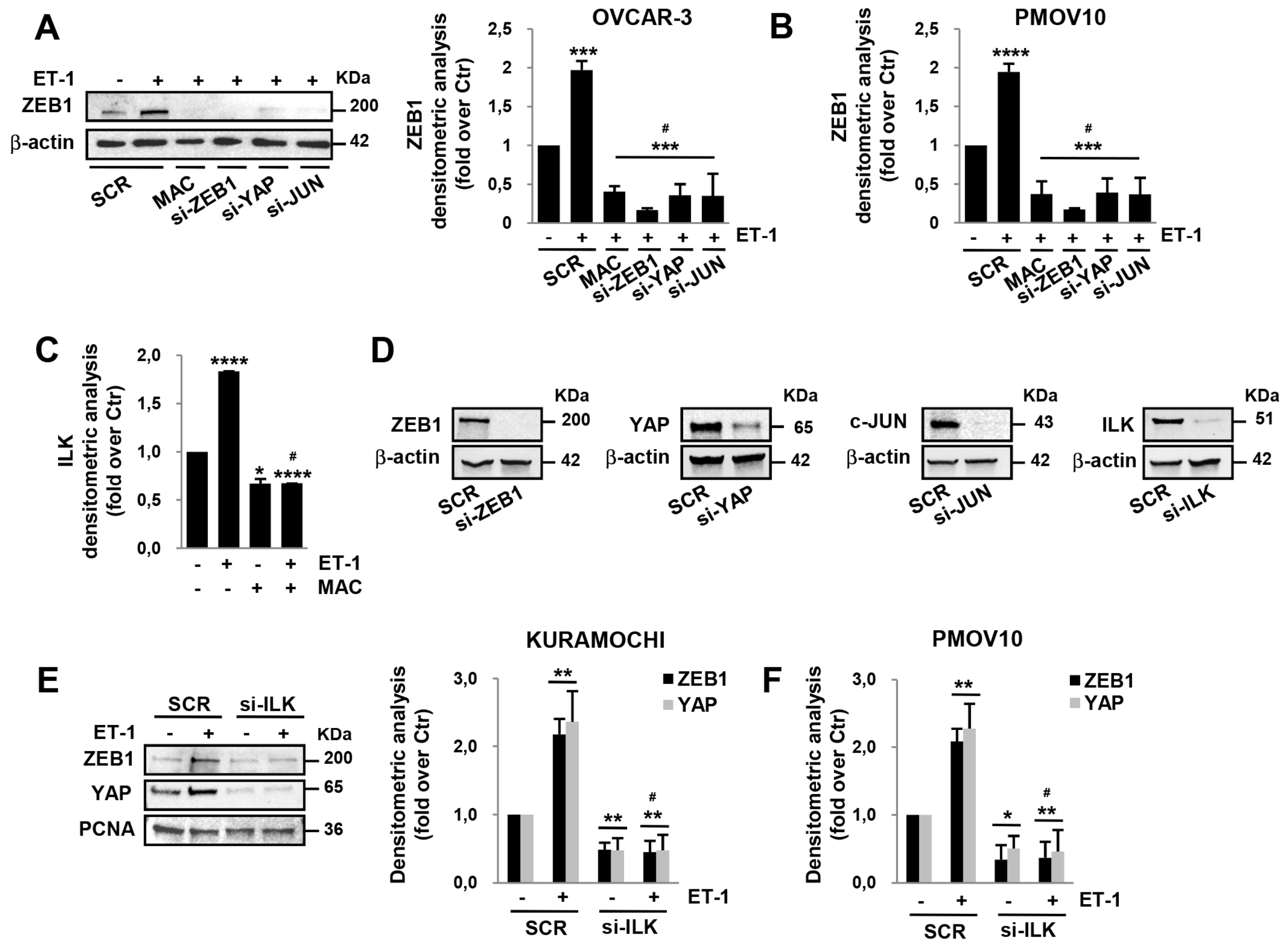


**Supplementary Figure S4.** YAP and AP-1 are involved in ET-1/ILK-triggered regulation of ZEB1. **A** Total extracts of OVCAR-3 cells transfected with SCR, si-ZEB1, si-YAP, or si-JUN for 72 h and stimulated with ET-1 and/or treated with macitentan for 48 h as indicated were IB with anti-ZEB1 Ab. Right graph represents the densitometric analysis of ZEB1 protein expression normalized to β-actin. Values are the mean ±SD expressed as fold induction (n=3; ****p*<0.001; #: *p* value was calculated vs. SCR+ET-1). **B** Densitometric analysis of ZEB1 protein expression normalized to β-actin of IB with total extracts of PMOV10 cells treated and transfected as in *A*. Values are the mean ±SD expressed as fold induction (n=3; ****p*<0.001; *****p*<0.0001; #: *p* value was calculated vs. SCR+ET-1). **C** Densitometric analysis of ILK protein expression normalized to β-actin of IB with total extracts of PMOV10 cells stimulated with ET-1 and/or treated with

macitentan for 30 min as indicated. Values are the mean ±SD expressed as fold induction (n=3; **p*<0.05; **** *p*<0.0001; #: *p* value was calculated vs. ET-1). **D** IB analyses for the indicated proteins in total extracts of Kuramochi cells transfected for 72 h with SCR, si-ZEB1, si-YAP, si-JUN, or si-ILK. β-actin was used as loading control. **E** Nuclear extracts of Kuramochi cells transfected with SCR or si-ILK for 72 h and stimulated with ET-1 for 6 h were IB for ZEB1 or YAP. Right graph represents the densitometric analysis of ZEB1 and YAP protein expression normalized to PCNA. Values are the mean ±SD expressed as fold induction (n=3; ***p*<0.01; #: *p* value was calculated vs. SCR+ET-1). **F** Densitometric analysis of ZEB1 and YAP protein expression normalized to PCNA of IB with nuclear extracts of PMOV10 cells treated and transfected as in *E*. Values are the mean ±SD expressed as fold induction (n=3; **p*<0.05; ***p*<0.01; #: *p* value was calculated vs. SCR+ET-1).

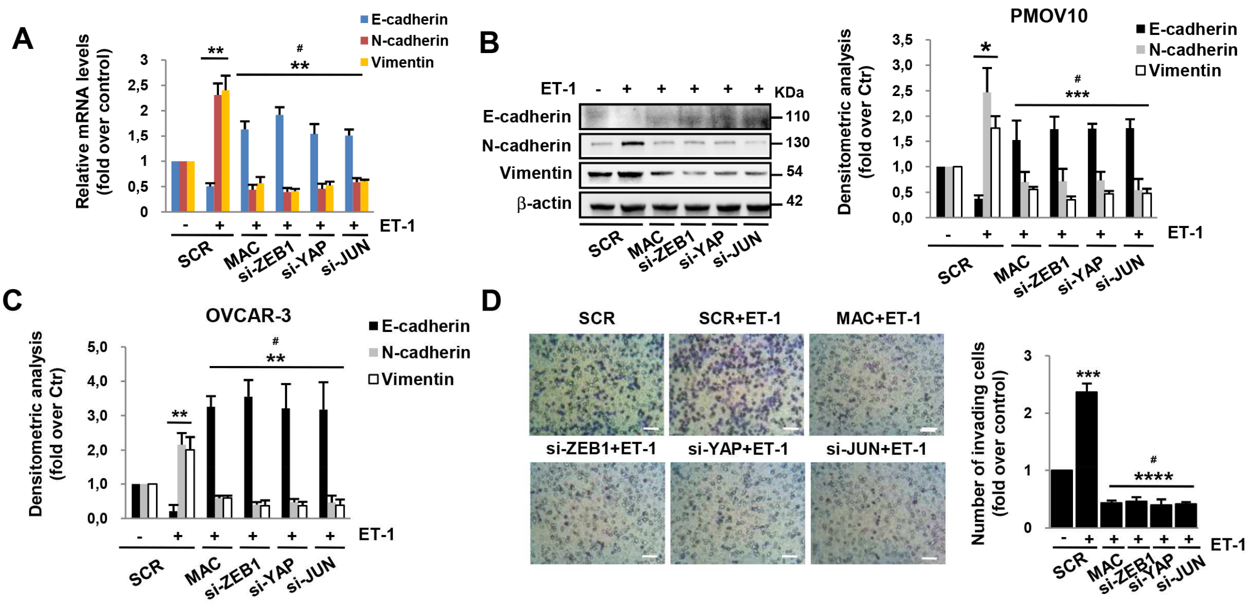


**Supplementary Figure S5.** YAP/AP-1/ZEB1 complex is involved in ET-1-induced EMT and cell invasion. **A** E-cadherin, N-cadherin and Vimentin gene expression in PMOV10 cells transfected with SCR, si-ZEB1, si-YAP, or si-JUN for 72 h and stimulated with ET-1 and/or treated with macitentan for 24 h as indicated was analyzed by q-RT-PCR and normalized to cyclophilin-A. Values are the means ± SD expressed as fold over control (n = 3; ***p*<0.01; #: *p* value was calculated vs. SCR+ET-1). **B** Total extracts of PMOV10 cells transfected for 72 h and stimulated for 48 h as in *A* were IB for the indicated proteins. Right graph represents the densitometric analysis of E-cadherin, N-cadherin, and Vimentin protein expression normalized to β-actin. Values are the mean ±SD expressed as fold induction (n = 3; **p*<0.05; ****p*<0.001; #: *p* value was calculated vs. SCR+ET-1). **C** Densitometric analysis of E-cadherin, N-cadherin, and Vimentin protein expression normalized to β-actin of IB with total extracts of OVCAR-3 cells stimulated and treated as in *B*. Values are the mean ±SD expressed as fold induction (n = 3; ***p*<0.01; #: *p* value was calculated vs. SCR+ET-1). **D** Transwell chemoinvasion assay with PMOV10 cells transfected as in *A* and overnight allowed to invade in presence of ET-1 and/or macitentan. Images represent the crystal violet-stained invasive cells (Magnification: 20x; scale bar: 100 μm). Right graph represents the

number of invading cells. Values are the means ± SD expressed as fold over control (n = 3; ***$p$<0.001; ****$p$<0.0001; #: $p$ value was calculated vs. SCR+ET-1).

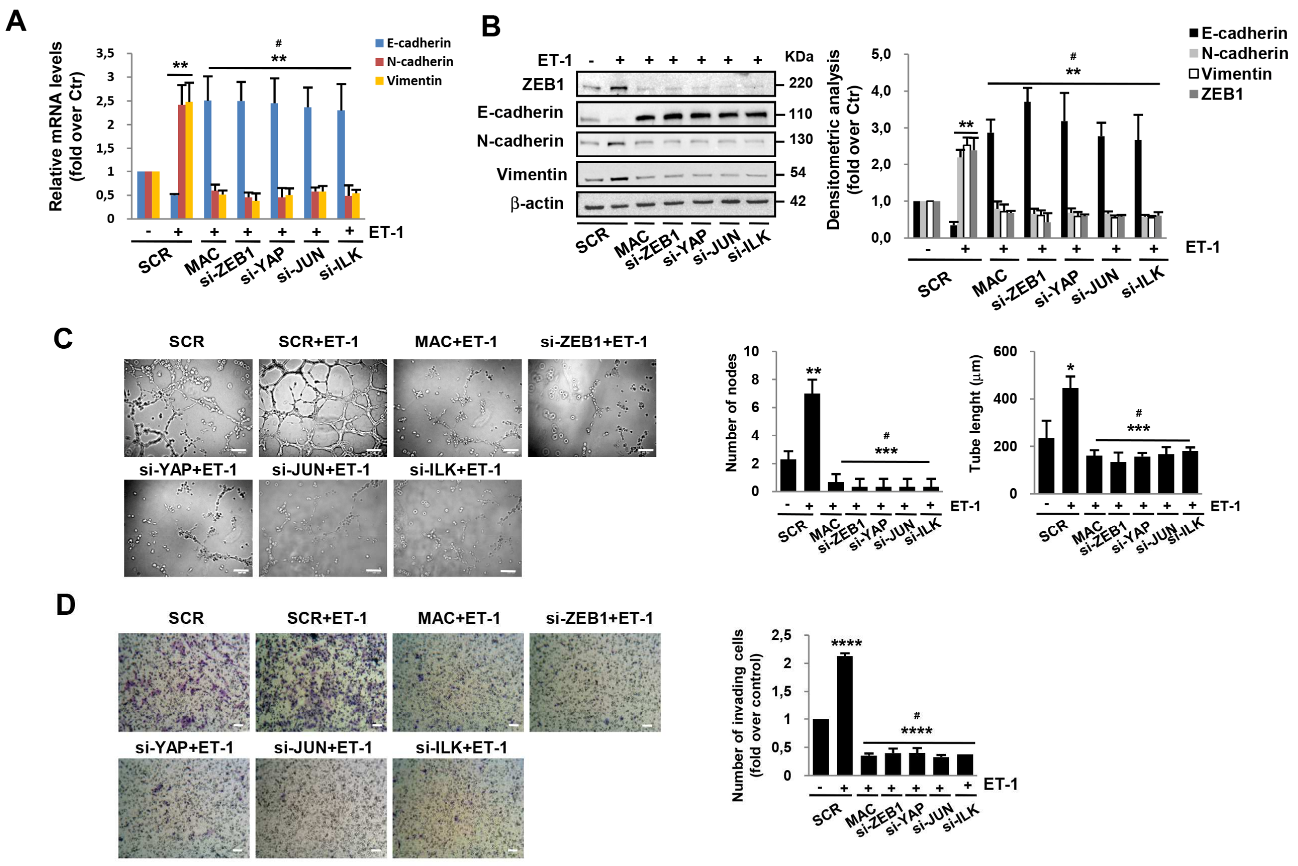


**Supplementary Figure S6.** ET-1 signaling drives ovarian cancer cell aggressiveness through the ILK/YAP/AP-1/ZEB1 network. **A** E-cadherin, N-cadherin and Vimentin gene expression in Kuramochi cells transfected with SCR, si-ZEB1, si-YAP, si-JUN, or si-ILK for 72 h and stimulated with ET-1 and/or treated with macitentan for 24 h as indicated was analyzed by q-RT-PCR and normalized to cyclophilin-A. Values are the means ± SD expressed as fold over control (n = 3; **$p$<0.01; #: $p$ value was calculated vs. SCR+ET-1). **B** Total extracts of Kuramochi cells transfected for 72 h and stimulated for 48 h as in *A* were IB for the indicated proteins. Right graph represents the densitometric analysis of ZEB1, E-cadherin, N-cadherin, and Vimentin protein expression normalized to β-actin. Values are the mean ±SD expressed as fold induction (n = 3; **$p$<0.01; #: $p$ value was calculated vs. SCR+ET-1). **C** Vasculogenic mimicry assay with

Kuramochi cells transfected for 48 h and overnight stimulated as in *A* (Magnification: 20x; scale bar: 100 μm). Right graphs represent the quantification of the number of nodes and the tube length. Columns show the mean ± SD (n = 3; *$p<0.05$; **$p<0.01$; ***$p<0.001$; #: $p$ value was calculated vs. SCR+ET-1). **D** Transwell chemoinvasion assay with Kuramochi cells transfected as in *A* and overnight allowed to invade in presence of ET-1 and/or macitentan. Images represent the crystal violet-stained invasive cells (Magnification: 10x; scale bar: 100 μm). Right graph represents the number of invading cells. Values are the means ± SD expressed as fold over control (n = 3; ****$p<0.0001$; #: $p$ value was calculated vs. SCR+ET-1).
